# BDNF and GDNF in Parkinson’s Disease: Associations with Clinical Features, Disease Course, and Progression—A Systematic Review

**DOI:** 10.1007/s12035-025-05649-z

**Published:** 2026-02-16

**Authors:** Julia Węgrzynek-Gallina, Aleksandra Buczek, Jakub Malkiewicz, Tomasz Chmiela, Tomasz Gallina, Patrycja Hudzińska, Joanna Siuda

**Affiliations:** 1 Department of Neurology, Faculty of Medical Sciences in Katowice, University Clinical Centre Prof K. Gibinski, Medical University of Silesia, 14 Medykow St, 40-752 Katowice, Poland; 2 Department of Neurology, Faculty of Medical Sciences in Katowice, Medical University of Silesia, 14 Medykow St, 40-752 Katowice, Poland; 3 Department of Cardiology and Structural Heart Disease, Faculty of Medical Sciences in Katowice, Medical University of Silesia, Upper-Silesian Medical Centre in Katowice, 45/47 Ziołowa St, Katowice, 40-635 Poland

**Keywords:** Parkinson’s disease, GDNF, BDNF, Neurotrophic factors, Biomarkers

## Abstract

Glial cell line-derived neurotrophic factor (GDNF) and brain-derived neurotrophic factor (BDNF) are proteins essential for neuronal survival and implicated in Parkinson’s disease (PD) pathophysiology. Although reduced levels of these neurotrophins have been observed in PD, their relationship with disease progression remains unclear. We conducted a systematic review by independently searching four databases using predefined keywords: Parkinson AND (GDNF OR BDNF OR neurotroph) AND (serum OR blood OR cerebrospinal fluid). After screening 2132 records, 35 studies qualified for inclusion. Changes in neurotrophic factors’ levels were evaluated in relation to disease severity and duration. Many studies reported a decline in BDNF levels associated with more severe motor symptoms. Some studies noted increased BDNF levels in advanced PD. This pattern may be affected by levodopa treatment, suggesting that elevated BDNF levels in advanced PD could reflect a treatment-related effect rather than disease progression itself. Reduced levels of both GDNF and BDNF were linked to cognitive decline, with BDNF also decreased in PD patients with depression. Serum BDNF levels were associated with motor severity and neuropsychiatric symptoms. BDNF levels in PD may increase with longer disease duration, likely due to levodopa treatment effects. However, lower BDNF levels are seen in cognitive decline and depression, frequent non-motor symptoms of PD. Further research is required to clarify BDNF dynamics and to determine GDNF’s role in motor progression and cognitive decline.

**Supplementary Information:**

The online version contains supplementary material available at 10.1007/s12035-025-05649-z.

## Background

Parkinson’s disease (PD) is one of the most prevalent neurodegenerative disorders, primarily characterized by motor symptoms such as bradykinesia, muscular rigidity, resting tremor, and postural instability, and several non-motor manifestations. Its pathophysiology involves neuroinflammation, oxidative stress, mitochondrial dysfunction, and the accumulation of alpha-synuclein aggregates, ultimately leading to the degeneration of dopaminergic neurons in the substantia nigra pars compacta, a brain region crucial for motor control [1].

Neurotrophic factors (NFs) are soluble polypeptides that play key roles in the development, growth, and functional regulation of neurons. They typically act via membrane-associated receptors with intrinsic tyrosine kinase activity, triggering the activation of transcription factors and the expression of genes essential for neuronal function [2]. Brain-derived neurotrophic factor (BDNF), expressed in multiple brain regions, plays a key role in neuronal differentiation, development, survival, synaptogenesis, and synaptic plasticity [3]. Another NF, glial-derived neurotrophic factor (GDNF), has a particular effect on dopaminergic neurons. It promotes the survival, morphological differentiation, damage repair, and dopamine release of these neurons, while also regulating their excitability in the midbrain [4, 5].

Current scientific evidence suggests that the levels of NFs, such as BDNF and GDNF, are decreased in neurodegenerative diseases, with several studies confirming this reduction in PD [6–9]. Both BDNF and GDNF have been investigated as potential biomarkers for specific clinical manifestations of PD. Altered levels of BDNF have been associated with motor symptom severity [3, 7, 10], but also with non-motor features such as depression and cognitive impairment [11, 12]. Similarly, GDNF may be associated with cognitive functions [13, 14]. Exploring the relationship between their concentrations and specific symptoms may provide significant knowledge about disease mechanisms and support the development of a more personalized therapeutic approach.

Previous systematic reviews and meta-analyses have primarily focused on selected aspects of Parkinson’s disease, particularly on the effects of physical exercise on BDNF levels, as well as comparisons between patients with PD and healthy controls [9, 15–17]. In the context of GDNF, available evidence is very limited, with the existing study primarily focusing on treatment-related aspects, providing little insight into its changes across the clinical course [18].

Consequently, significant gaps remain concerning how BDNF and GDNF levels change throughout the disease, including their relationship with motor severity, specific symptoms, and other clinical features. Given the central roles of BDNF and GDNF in neuronal survival and neuroprotection, and their extensive investigation in PD relative to other neurotrophic factors, this review focuses exclusively on these two. This targeted approach aims to clarify unresolved controversies and gaps, such as the dynamic behavior of BDNF levels throughout disease progression and the less well-characterized role of GDNF.

Therefore, our aim was to evaluate and summarize the available evidence on the relationship between BDNF and GDNF levels in body fluids and the clinical manifestations of PD, focusing on their potential utility as biomarkers for symptom profiling and disease monitoring.

## Material and Methods

This is a systematic review that analyzed studies on how the clinical presentation and progression of PD affect GDNF and BDNF levels. It examined changes in these NFs concerning disease severity, duration, and both motor and non-motor symptoms, including cognitive decline, mood disturbances, and autonomic dysfunction. The review focused on studies evaluating GDNF and BDNF as potential biomarkers of disease course.

In this study, the PRISMA (Preferred Reporting Items for Systematic Reviews and Meta-Analyses) guidelines [19] were followed to ensure transparency and rigor in the literature search, study selection, quality assessment, and data synthesis (see Online Resource 1 – PRISMA Checklist).

A systematic search of four databases (PubMed, Scopus, Web of Science, Embase) was conducted by two independent authors (JW-G and TG) using predefined criteria. Any disagreements were resolved through discussion. The search strategy included the following key terms: *Parkinson* AND (*GDNF* OR *BDNF* OR *neurotroph*) AND (*serum* OR *blood* OR *cerebrospinal fluid*). No restrictions were applied regarding the publication date. The search was conducted on November 12, 2025.

The selection criteria included original observational studies published in English. Reviews, case reports, case series, and conference abstracts were excluded. The detailed selection criteria are presented in Table 1.

**Table 1:** Selection criteria for the systematic review assessing the impact of the clinical presentation and progression of Parkinson’s disease on GDNF and BDNF levels

Inclusion criteria	Exclusion criteria
Studies including Parkinson’s disease patients or allowing for the extraction of data specific to this population	Research presenting other outcomes or a wrong design
Research evaluating changes in these BDNF and GDNF concerning disease severity, duration, both motor and non-motor symptoms, including cognitive decline, mood disturbances, and autonomic dysfunction	Research types of case studies, case series, reviews, editorials, conference abstracts, books, opinion articles, etc.
Studies evaluating BDNF and GDNF levels in blood, plasma, serum, or other morphological elements, or in cerebrospinal fluid	Experimental studies
Observational studies (case-control, cross-sectional, and cohort studies)	Animal studies
Articles available in English	Articles in other languages

The methodological quality of included studies was assessed using the Newcastle-Ottawa Scale (NOS) for Assessing the Quality of Nonrandomized Studies in Meta-Analyses [20]. It was performed independently by two authors (JW-G and TG). A detailed assessment was described in the “Results” section and the “Quality assessment of included studies” section, as well as in the table presented in Online Resource 2.

The screening and selection of articles were managed using the Rayyan software [21] to facilitate independent review and conflict resolution. This review aimed to collect and summarize the available knowledge on BDNF and GDNF levels across the course of Parkinson’s disease and the factors that may influence them. Due to the considerable heterogeneity in study designs, populations, and outcome measures, a meta-analysis was not possible to perform. Instead, a qualitative analysis of the data was conducted, including a narrative synthesis structured around clinical domains such as age and disease duration, motor symptoms, mood disorders, cognitive dysfunction, and other non-motor and metabolic aspects. This review was not registered; however, all stages of the review were conducted with the highest methodological rigor, following predefined eligibility criteria and procedures to ensure transparency and minimize the risk of bias.

**Fig. 1:**
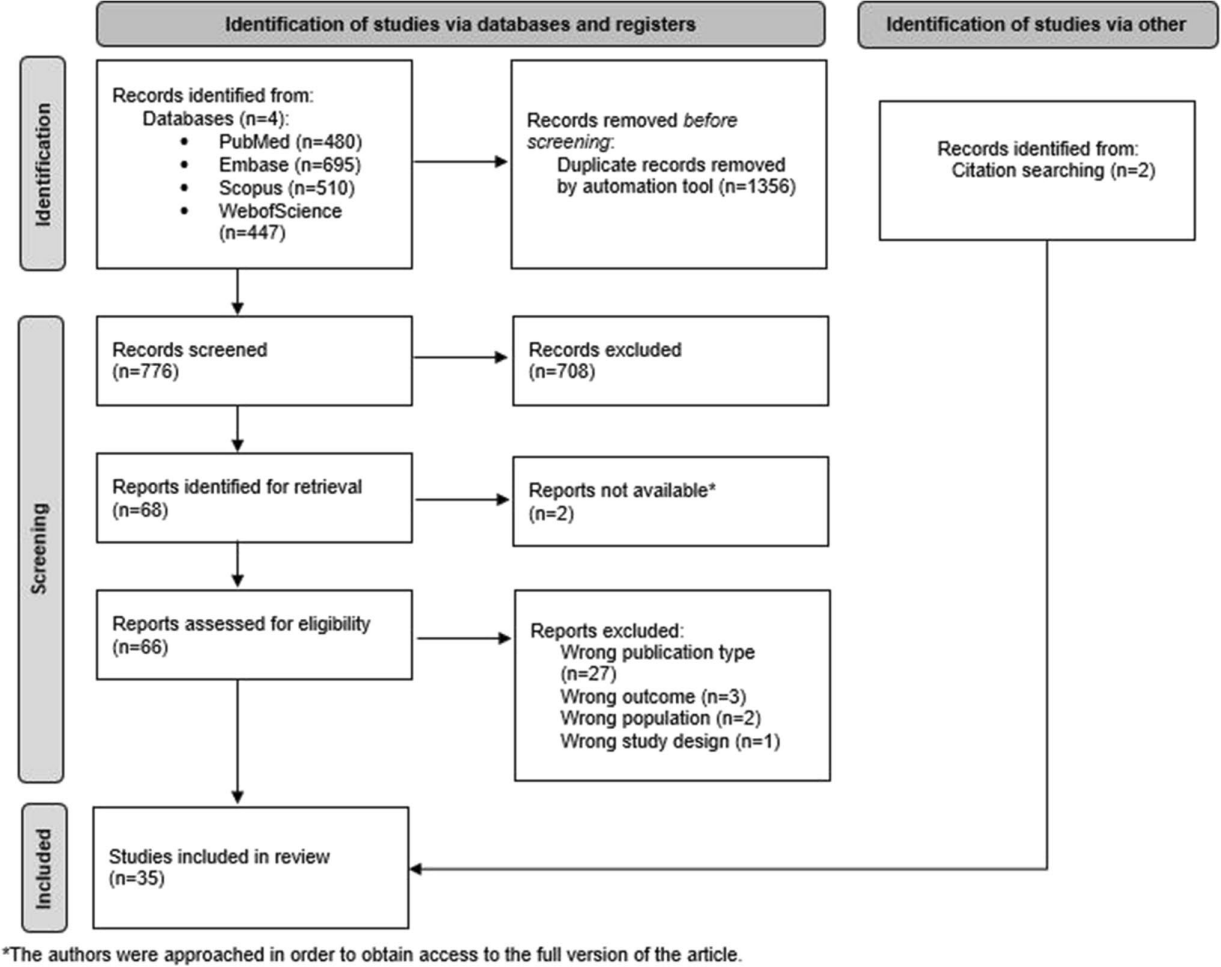
The detailed process of article selection in the systematic review assessing the impact of the clinical presentation and progression of Parkinson’s disease on GDNF and BDNF levels in biological samples is illustrated on the PRISMA diagram

Initially, 2132 records were identified. Duplicates were removed using an automated tool. A total of 776 articles were screened based on their title and abstract, of which 68 met the predefined criteria. Additionally, citation searching was conducted within the eligible articles, identifying 1 more study that met the inclusion criteria. Ultimately, 35 articles were included in the final analysis. Data from the included studies were synthesized into tables by two authors (AB and PH) and verified by a third author (JW-G). All qualified articles were screened for more studies evaluating the topic. Studies excluded after full-text review, along with the reasons for exclusion, are listed in Online Resource 3. A detailed flow of study selection, including the number of excluded and included articles, is illustrated in Fig. 1.

## Results

The final analysis included 35 observational studies, including 28 case-control studies, 5 cross-sectional studies, and 2 cohort studies conducted between 2009 and 2025.

Of these, 27 studies evaluated BDNF, 7 assessed GDNF, and 1 study analyzed both BDNF and GDNF. Regarding the biological material used, 29 studies measured neurotrophins’ levels in serum, 2 in plasma, 2 in cerebrospinal fluid (CSF), 1 in peripheral blood lymphocytes, and 1 in plasma-derived exosomes.

The studies included 2704 patients with PD in different stages of the disease, with an average sample size of 77.3 ± 62.2 participants (range, 13–259). All studies provided data on patients’ age; 34 reported the male-to-female ratio; 30 included disease duration; 11 reported the age of onset; 30 described the Hoehn-Yahr (H-Y) scale; and 28 assessed the Movement Disorder Society-Unified Parkinson’s Disease Rating Scale (MDS-UPDRS) or Unified Parkinson’s Disease Rating Scale (UPDRS) part III. Additionally, 21 studies specified the clinical state of patients during the examination. Antiparkinsonian treatment data were available in 29 studies: 27 included patients receiving treatment, while 2 involved untreated patients’ groups. Among the studies with treated patients, 10 reported the levodopa equivalent daily dose (LEDD). Details are shown in Table 2. *

**Table 2:** Characteristics of studies included in the systematic review on the relationship between BDNF, GDNF, and Parkinson’s disease

Number	Reference	Study type	Population	Material for laboratory tests	N enrolled at baseline (all patients)	N PD patients	N women at baseline (PD)	N men at baseline (PD)
1	Di Lazzaro et al. 2024 [22]	Cross-sectional study	PD-short disease duration - 35PD-long disease duration - 69	Serum	PD-short disease duration: 35PD-long disease duration: 69	104	45	59
2	Wang X et al. 2024 [11]	Cross-sectional study	PD depressionPD non depression	Serum	PD with depression: 108PD without depression: 108	216	PD with depression: 62PD without depression: 50	PD with depression: 46PD without depression: 58
3	Liu et al. 2024 [4]	Case-control study	PD-N, PD-MCI, PD-D, HC	Serum	PD-N: 52PD-MCI: 53HC: 45	105	PD-N: 5PD-MCI: 6	PD-N: 5PD-MCI: 4
4	Korkmaz et al. 2024 [42]	Case-control study	Patients with neurological diseases (epilepsy, AD, PD, MS), HC	Serum	PD: 15Epilepsy: 15AD: 15MS: 15HC: 15	15	9	6
5	Jin et al. 2023 [39]	Case-control study	PD-nRBD, PD-RBD, HC	Serum	PD-nRBD: 56 PD-RBD:45 HC: 53	101	PD-nRBD: 25 PD-RBD: 19	PD-nRBD: 31 PD-RBD: 26
6	Tang et al. 2023 [36]	Case-control study	PD-high-GDNFPD-low-GDNFHC	Serum	PD-high-GDNF: 19PD-low-GDNF: 19HC: 25	38	PD-high-GDNF: 8PD-low-GDNF: 12	PD-high-GDNF: 11PD-low-GDNF: 7
7	Badr et al. 2023 [32]	Case-control study	PD, HC	Serum	PD: 58HC: 45	58	20	38
8	Tong et al. 2023 [13]	Case-control study	PD-NPD-MCIPD-DHC	Serum	PD-N: 44PD-MCI: 41PD-D: 20	105	PD-N: 17PD-MCI: 18PD-D: 14	PD-N: 27PD-MCI: 23PD-D: 6
9	Wang et al. 2023 [38]	Case-control study	PD-SD, PD-NSD, HC	Serum	PD-NSD: 36PD-SD: 51HC: 49	87	PD-NSD: 19PD-SD: 24	PD-NSD: 17PD-SD: 27
10	Kaminska et al. 2022 [40]	Cohort study	PD (OSA-, OSA+CPAP+, OSA+CPAP-)	Serum	PD without OSA: 20PD with OSA and CPAP: 22PD with OSA and without CPAP: 24	66	PD without OSA: 8PD with OSA and CPAP: 7PD with OSA and without CPAP: 10	OSA-: 12, OSA+CPAP+: 15, OSA+CPAP-: 14PD without OSA: 12PD with OSA and CPAP: 15PD with OSA and without CPAP: 14
11	Chen et al. 2022 [41]	Cross-sectional study	Cons-Pro-PD, nCons-PD, Cons-clinic-PD	Serum	Cons-Pro-PD: 48nCons-PD: 49Cons-clinic-PD: 31	128	Cons-Pro-PD: 19, nCons-PD: 24, Cons-clinic-PD: 13Cons-Pro-PD: 19nCons-PD: 24Cons-clinic-PD: 13	Cons-Pro-PD: 29nCons-PD: 25Cons-clinic-PD: 18
12	Alomari et al. 2022 [43]	Case-control study	PD, HC	Plasma	PD: 24HC: 27	24	9	15
13	Roy et al. 2021 [7]	Case-control study	PD	Serum	PD: 27HC: 15	27	13	14
14	Yi et al. 2021 [25]	Cohort study	Ex-PD, Ex-NPD, Po-PD, Po-NPD	Serum	ex-PD group: 111ex-NPD group: 45	156	ex-PD - 46 ex-NPD - 24	ex-PD - 65ex-NPD- 21
15	Shi et al. 2021 [14]	Case-control study	PD-CI, PD-N, HC	Serum	PD-N: 26PD-CI: 27HC: 26	53	PD-N: 13PD-CI: 17	PD-N: 13PD-CI: 10
16	Ekmekyapar et al. 2021 [26]	Case-control study	PD (PD-MCI, PD-Mild dementia, PD- Moderate dementia) HC	Serum	PD-MCI: 36PD with mild dementia: 19PD with moderate dementia: 8HC: 33	63	29	34
17	Huang et al. 2021 [24]	Case-control study	PD with RLS, PD without RLS, HC without RLS, HC with RLS	Serum	PD with RLS: 53PD without RLS: 196 Control with RLS:24Control without RLS: 302	249	PD with RLS: 30PD without RLS: 94	PD with RLS: 23 PD without RLS: 102
18	Chung et al. 2020 [3]	Case-control study	PD	Plasma exosomes	PD: 114HC:42	114	52	62
19	Huang et al. 2021 [12]	Case-control study	PD with depression, PD without depression, controls	Serum	PD with depression:122PD without depression: 137	259	122	137
20	Huang et al. 2019 [10]	Case-control study	PD	Peripheral blood lymphocytes	PD: 28ET: 28HC: 28	28	12	16
21	Liu et al. 2020 [37]	Case-control study	PD-MCI, PD-N, PD-D, controls	Serum	PD-N: 44PD-MCI: 41PD-D: 20HC: 43	105	43	69
22	Rocha et al. 2018 [2]	Case-control study	PD	Serum	PD: 40HC: 25	40	13	27
23	Huang et al. 2018 [23]	Case-control study	PD	Serum	PD: 60ET: 60HC: 60	60	28	32
24	Alomari et al. 2018 [44]	Case-control study	PD, controls	Plasma	PD: 28HC: 30	28	no data	no data
25	Wang et al. 2017 [27]	Case-control study	PD with depression, PD without depression,	Serum	PD with depression: 46PD without depression: 50HC: 102	96	PD with depression: 22PD without depression: 19	PD with depression: 24PD without depression: 31
26	Siuda et al. 2017 [30]	Case-control study	AD, MCI, PD without cognitive impairment, HC	Serum	PD: 49MCI: 115AD: 134HC: 80	49	22	27
27	Wang et al. 2016 [8]	Case-control study	PD, controls	Serum	PD: 97HC: 102	97	43	54
28	Costa et al. 2015 [33]	Case-control study	PD, controls	Serum	PD: 13HC: 20	13	no data	no data
29	Khalil et al. 2015 [34]	Case-control study	PD, controls	Serum	PD: 29	29	12	17
30	Ventriglia et al. 2013 [28]	Case-control study	PD	Serum	PD: 30VAD: 91LBD: 40FTD: 28AD: 266HC: 169	30	9	21
31	Ziebell et al. 2012 [45]	Cross-sectional study	Patients with positive DAT-scan results	Serum	Patients with positive DaT-Scan results: 21	21	5	16
32	Leverenz et al. 2011 [35]	Cross-sectional study	PD without dementia	CSF	PD: 22	22	5	17
33	Pålhagen et al. 2010 [31]	Case-control study	PD, PD + MD, MD (major depression)	CSF	PD without depression: 14PD with major depression: 11	25	PD without depression: 6PD with major depression: 5	PD without depression: 8PD with major depression: 6
34	Scalzo et al. 2010 [6]	Case-control study	PD	Serum	PD: 47HC: 23	47	23	8
35	Ricci et al. 2010 [29]	Case-control study	PD, HC	Serum	PD depressed: 26PD non depressed: 20HC: 14	46	PD depressed: 12PD non depressed:8	PD depressed: 14PD non depressed: 12

Number	Age of patients (years)	Disease duration at baseline (years)	Age of onset (years)	H-Y Scale	Mean/median UPDRS III at baseline	State during examination(ON/OFF/No data)	Antiparkinsonian treatment	Mean/median baseline LEDD (mg)
1	66 ± 9.55	8 ± 5	59 ± 10	no data	22.2 ± 13	ON	yes	581 ± 403
2	PD with depression - female: 65.56±7.07PD with depression - male: 67.52±7.00PD without depression - female: 65.32±6.24PD without depression -male: 64.31±6.66	PD with depression - female: 4.17±2.88PD with depression - male: 5.05±4.11PD without depression - female: 3.74±2.99PD without depression -male: 4.35± 3.81	PD with depression - female: 60.89±7.21PD with depression - male: 63.66±7.36PD without depression - female: 61.65±5.85PD without depression -male: 59.78±7.06	Range 1–4	PD with depression - female: 41.44±19.62PD with depression - male: 44.04±18.63PD without depression - female: 26.86±14.62PD without depression -male: 32.72±16.50	OFF	yes	no data
3	PD-N: 64.45±8.15PD-MCI: 64.02±9.70	PD-N: 24 [11, 60]PD-MCI: 24 [12, 72]	no data	PD-N: 1.75 [1, 2]PD-MCI: 2.5 [2, 2.5]	PD-N: 19.32 ± 7.93PD-MCI: 25.37 ± 9.00	ON	yes	no data
4	PD: 65.73±9.316	no data	no data	no data	no data	no data	no data	no data
5	PD-nRBD: 58.5 [52.5, 60] PD-RBD: 63 [59, 69]	PD-nRBD: 3.75 [2,5] PD-RBD: 4 [3, 6]	no data	PD-nRBD: 2 [1.5, 2.5] PD-RBD: 2 [1.5, 2.5]	PD-nRBD: 19 [12.75, 27.25] PD-RBD: 25 [19, 34]	OFF	yes	PD-nRBD: 405 (300, 631.25] PD-RBD: 405 [300, 631.25]
6	PD-high-GDNF: 62.16±9.714PD-low-GDNF: 65.26±4.458	PD-high-GDNF: 3.421±1.169PD-low-GDNF: 5.657±3.077	no data	median [IQR]PD-high-GDNF: 1.5 [1, 2]PD-low-GDNF: 2 [1.5, 3]	no data	no data	yes	PD-high-GDNF: 200±70.71PD-low-GDNF: 263.16±94.78
7	58.448±4.87	3.000±1.05	no data	1.526±0.517	26.086 ± 2.28	ON	yes	no data
8	PD-N: 61.66 ± 8.31PD-MCI: 65.02 ± 8.61PD-D: 67.75 ± 6.16	median, monthsPD-N: 24 [9, 57] PD-MCI: 24 [12, 66]PD-D: 54 [24, 93]	no data	PD-N: 1.75 [1, 2] PD-MCI: 2.5 [2, 2.75]PD-D: 3 [2.5, 3]	PD-N: 20.24 ± 8.03 PD-MCI: 28.24 ± 8.88 PD-D: 34.08 ± 7.28	ON	yes	PD-N: 297.16 ± 27.48 PD-MCI: 354.57 ± 31.37 PD-D: 534.38 ± 56.19
9	PD-NSD: 66.86 ± 8.9PD-SD: 67.57 ± 8.75	PD-NSD: 54.00 [26.75, 72.00] months; PD-SD: 66.00 [48.00, 82.00] months	no data	PD-NSD: 2.00 [1.00, 2.50]PD-SD: 2.50 [2.00, 3.00]	PD-NSD: 22.67 ± 12.97PD-SD: 30.98 ± 16.26	ON	yes	PD-NSD: 412.50 [337.50, 596.88]PD-SD: 425.00 [337.50, 575.00]
10	PD without OSA: 61.2±8.3PD with OSA and CPAP: 66.2±8.6PD with OSA and without CPAP: 65.8±11.6	no data	no data	no data	PD without OSA: 15.9.2±7.2PD with OSA and CPAP: 23.3.2±13.9PD with OSA and without CPAP: 25.8.8±13.7	no data	no data	no data
11	Cons-Pro-PD: 68.35 ± 8.353nCons-PD: 64.73 ± 9.565Cons-clinic-PD: 70.52 ± 8.733	Cons-Pro-PD: 48 [24 84]nCons-PD: 36 [15.5, 60]Cons-clinic-PD: 88 [48, 125] [month]	Cons-Pro-PD: 63.66 ± 8.88 nCons-PD: 60.18 ± 10.40 Cons-clinic-PD: 61.53 ± 10.39	Cons-Pro-PD: 2 [2, 3]nCons-PD: 2 [1, 3] Cons-clinic-PD: 3 [2, 4]	Cons-Pro-PD: 36 [22, 58]nCons-PD: 30 [20, 48]Cons-clinic-PD: 48 [33, 80]	ON	yes	Cons-Pro-PD: 443.75 [162.5, 600]nCons-PD: 337.5 [37.5, 400] Cons-clinic-PD: 537.5 [400, 637.5]
12	56.5±13.0	no data	no data	1.4±0.5	54.3±29.1	no data	yes	no data
13	63± 8	6.72 ±3.36	55.76 ±8.48	2.0 ±0.82	25.76 ±15.09	no data	yes	763.41±66.57
14	ex-PD 61.1 ± 8.8ex-NPD 60.0 ± 9.2	newly diagnosed (<1y)	no data	<2.5	no data	no data	no	no treatment
15	PD-N: 65.04±10.55PD-CI: 68.07±6.81	median, monthsPDN 24 [IQR31.5], PDCI 48 [74]	no data	PD-N: 1[IQR:1]PDCI: 2 [IQR1.5]	no data	no data	no data	no data
16	Whole PD group: 65.54 ± 7.07	Whole PD group: 5.32±3.75	Whole PD group: 60.13±7.12	Range: 1–3	PD-MCI: 22.47 ± 13.23Pd with mild dementia: 25.94 ± 12.43PD with moderate dementia: 32.62 ± 12.92	no data	no data	no data
17	PD with RLS: 62.81±9.94PD without RLS: 61.72±9.17	PD with RLS: 4.72±2.74PD without RLS: 4.25±2.33	PD with RLS: 58.09±8.67PD without RLS: 57.47±8.25	PD with RLS: 1.75±0.94PD without RLS: 1.56±0.78	PD with RLS: 41.34±15.53PD without RLS: 37.47±14.05**	ON	yes	PD with RLS: 448.1 ±190.03PD without RLS: 410.15±181.09
18	69.67 ± 8.44	2.70 ± 2.45	no data	no data	22.89 ± 10.00	ON	yes	no data
19	PD with depression: 62.84±8.73PD without depression: 62.19±9.51	PD with depression: 4.96±2.09PD without depression: 4.37±1.74	PD with depression: 57.89 ± 9.13PD without depression: 57.82 ± 9.56	PD with depression: 2.07±1.02PD without depression: 1.62±0.79	PD with depression: 33.78 ± 8.31PD without depression: 29.51 ± 7.15	ON	yes	no data
20	62.5 ± 10.4	4.5 ± 3.18	no data	2.32±1.39	43.64 ± 33.34**	ON/OFF*	yes (14.5% of patients)	no data
21	PD-N: 63.45±8.15PD-MCI: 65.02±9.70PD-D: 68.30±6.72	median, monthsPD-N: 24 [11, 60]PD-MCI: 24 [12, 72]PD-D: 36 [24, 96]	no data	PD-N: 1.75 [1, 2]PD-MCI: 2 [2, 2.5]PD-D 3 [2.5, 3]	PD-N: 19.32±7.93PD-MCI: 25.37±9.00PD-D: 30.98±9.45	no data	no data	no data
22	68.71±10.07	5.45±4.13	no data	2.44±0.69	34.56 ± 18.43	ON	yes	no data
23	62.5±9.9	6.6±4.0	55.9±10.8	1.9±1.1	44.0 ± 31.1**	no data	yes	no data
24	59.4±13.1	4.4±2.7	no data	2.4±0.7	49.2±16.5	ON	yes	no data
25	PD with depression: 63.85 ± 9.50PD without depression: 61.64 ± 8.87	PD with depression: 4.63 ± 3.53PD without depression: 3.76 ± 2.56	PD with depression: 60.96 ± 10.20PD without depression: 57.58 ± 8.00	PD with depression: 1.72 ± 0.69PD without depression: 1.63 ± 0.70	no data	ON	yes	no data
26	63.3±10.5	8.41±5.8	no data	2.7±0.7	no data	no data	yes	940.2±493.8
27	63.60 ± 9.32	4.23 ± 3.10	no data	1.59 ± 0.43	no data	no data	yes	no data
28	68.3± 7.8	8.8 ± 6.9	no data	no data	26.5±11.1	ON	yes	695± 294
29	59.4±13.1	4.4 ± 2.7	no data	2.4 ± 0.7	49.2 ± 16.5	ON	yes	no data
30	67.6±8.4	no data	no data	3.58 ± 0.5	no data	ON	yes	no data
31	70.0 [62, 88]	28.9 [6–96] months	no data	2.8 [1.5, 4]	46.1 [19, 67]**	OFF	no (20/21)	no data
32	68.7± 8.0	no data	59.2±11.6	2.5±0.8	21±9**	no data	no data	no data
33	PD without depression: 65.3±7.2 PD with major depression: 64.3±10.1	PD without depression: 6.9±2.4PD with major depression: 9.7±4.7	no data	PD without depression: 1.8±0.4PD with major depression: 2.2±0.4	PD without depression: 22.6±10.1PD with major depression: 24.9±10.8	OFF	yes	no data
34	65.7±8.8	7.6 ± 4.5	58.5 ± 9.7	median 2, range:1–4	34.5 ± 22.3	no data	yes (33/47)	no data
35	PD depressed: 63.73±7PD non depressed: 63.7±8.87	PD depressed: 8.76±2.17PD non depressed: 8.05 ±2.41	no data	PD depressed: 2.03 ±0.72PD non depressed: 1.7 ±0.65	PD depressed: 21.96 ±8.56PD non depressed: 19.65±7.31	ON	yes	no data

Values are expressed as mean ± SD, median [IQR or Q1, Q3]

*PD* Parkinson’s disease, *BDNF* brain-derived neurotrophic factor, *GDNF* glial-derived neurotrophic factor, *LEDD* levodopa equivalent daily dose, *MCI* mild cognitive impairment, *PD–N* Parkinson’s disease cognitive normal, *AD* Alzheimer’s disease, *MS* multiple sclerosis, *HC* healthy controls, *ET* essential tremor, *FTD* frontotemporal dementia, *VAD* vascular dementia, *LBD* dementia with Lewy bodies, *PD-low-GDNF* Parkinson’s disease with low serum GDNF, *PD-high-GDNF* Parkinson’s disease with high serum GDNF, *PD-SD* PD with sleep disturbance, *PD-NSD* PD without sleep disturbance, *Cons-clinic-PD* Parkinson’s disease with clinical stage constipation, *Cons-Pro-PD* Parkinson’s disease with prodromal stage constipation, *nCons-PD* Parkinson’s disease without constipation, *PD-NSD* Parkinson’s disease without sleep disorders, *PD-SD* Parkinson’s disease with sleep disorders, *PD with OSA and CPAP* Parkinson’s disease with obstructive sleep apnea who are using continuous positive airway pressure treatment, *PD with OSA without CPAP* Parkinson’s disease with obstructive sleep apnea who are not using continuous positive airway pressure treatment, *PD with RLS* Parkinson’s disease with restless legs syndrome, *PD without RLS* Parkinson’s disease without restless legs syndrome, *DaT-Scan* dopamine transporter scan, *PD-nRBD* PD without rapid eye movement (REM) sleep behavior disorder, *PD-RBD* PD with REM sleep behavior disorder

*Partial treatment administration

**UPDRS total score

All included studies were categorized according to the type of associations analyzed: age and disease duration, motor symptoms, neuropsychiatric symptoms, other non-motor symptoms, and metabolic aspects, with appropriate subsections. The results were presented in a narrative format, and the main direction of findings is illustrated in Table 3. The full detailed data was provided in Suppl. Tables titled Online Resource 4, 5, 6, 7, 8, 9.

**Table 3:** Summary of evidence on associations between BDNF and GDNF and clinical aspects of PD

Domain	Neurotrophic factor	Number of studies	Main direction of findings	Consistency
Age of patients	BDNF	1	↓ BDNF with higher age [22]	Insufficient to assess
Age of onset	BDNF	2	↓ BDNF with later age of onset [23], no change in BDNF [24]	Inconsistent
Disease duration	BDNF	7	↑ BDNF with longer disease duration [6, 10, 23], ↓ BDNF with longer disease duration [22, 24], ↓ mBDNF, proBDNF with longer disease duration [25], no change in BDNF, proBDNF [2]	Inconsistent
	GDNF	1	No change in GDNF [2]	Insufficient to assess
Motor symptoms		13	↓ BDNF with severity [6, 7, 12], ↓ BDNF in more severe axial symptoms [3], ↑ BDNF in tremor-dominant PD [22], ↑ BDNF with severity [10, 23], no change in BDNF [2, 8, 24, 26–28], no change in pro-BDNF [2]	Inconsistent
Mood disorder	BDNF	7	↓ BDNF in depressed PD [12, 27, 29], no change [6, 30–32]	Inconsistent
	GDNF	1	No change [29]	Insufficient to assess
Cognitive impairment	BDNF	8	↓ BDNF [8, 11, 33, 34], no change [6, 26, 34, 35]	Inconsistent
	GDNF	5	↓ GDNF in cognitive impairment [4, 13, 14, 36, 37]	Consistent
Other non-motor symptoms	BDNF	3	↓ BDNF in RLS [38] ↓ BDNF in RBD [39] ↑ BDNF in daytime sleepiness [40]	Limited, but partly consistent
	GDNF	2	↓ GDNF in sleep disorders [24] ↓ GDNF in constipation [41]	Limited, but consistent
Metabolic effects	BDNF	4	No clear associations [26, 42]; single links with lipids [43] and preserving vascular function [44]	Insufficient to assess

*PD* Parkinson’s disease, *BDNF* brain-derived neurotrophic factor, *GDNF* glial-derived neurotrophic factor, *RLS* restless legs syndrome, *RBD* rapid eye movement sleep behavior disorder

### Age and Disease Duration

A limited number of studies addressed age-related aspects of BDNF expression [22–24]. Di Lazzaro reported a negative correlation between BDNF levels and patients’ age [22]. Huang and Yun observed that BDNF levels decreased with later age at disease onset [23]. On the other hand, Huang et al. found no significant association between age at onset and BDNF concentration in serum [24]. In summary, evidence regarding age-related changes in BDNF levels is limited and inconsistent.

There is more data regarding disease duration. Huang et al. found positive correlations between BDNF levels in peripheral blood lymphocytes and disease duration [10]. Similar results were gained by Scalzo et al. and Huang et al. in 2018 in serum [6, 23]. In 2021, Huang et al. observed negative correlations with disease duration, indicating that BDNF decreases with greater length of illness [24]. Di Lazzaro et al. noted a negative correlation between serum BDNF levels and disease duration as well. They did not observe a statistically significant difference between patients with shorter (< 5 years) and longer (> 5 years) disease duration either [22]. Yi et al. observed that individuals with prodromal Parkinson’s disease had higher proBDNF, lower mBDNF, and reduced mBDNF/proBDNF ratios at baseline and 1-year follow-up. The mBDNF/proBDNF ratio showed the highest diagnostic value for early PD, suggesting altered BDNF processing in early disease stages [25]. Rocha et al. found no associations between BDNF, pro-BDNF, GDNF, and disease duration [2]. All detailed results are described in the Online Resource 4 (Suppl. Table 4).

### Motor Symptoms

Thirteen studies investigated the association between NFs’ levels and motor symptoms. Twelve of these studies focused on BDNF, while one examined both BDNF and GDNF.

Motor severity was assessed using scales such as the H-Y scale, the UPDRS/MDS-UPDRS parts I, II, and III, as well as more specific functional assessments like the Timed Up and Go (TUG) test, the 6-Minute Walk Test (6-MWT), the Berg Balance Scale (BBS), or Comfortable Gait Speed (CGS).

Several studies reported a decrease in serum BDNF levels with greater motor dysfunction severity [3, 7, 24]. Roy et al. observed that BDNF decreased with greater motor impairment measured in the UPDRS part III score (*r* = −0.823, *p* = 0.001) as well as on the H-Y Scale (H-Y I vs. H-Y III, *p* = 0.0001) [7]. Similar findings were reported by Huang et al. [12]. Chung et al. reported that lower plasma exosomal BDNF levels correlated with more severe axial motor symptoms, such as postural instability and gait impairment, as assessed by the UPDRS parts I and III [3]. Scalzo et al. found consistent results, showing reduced serum levels of BDNF associated with poor balance on the BBS, longer times on the TUG, reduced gait speed, and shorter distances covered in the 6MWT [6]. Huang et al. in 2018 and 2019 found positive correlations between BDNF levels and H-Y, UPDRS. These authors also found that BDNF increased only in early stages (H-Y I and II) and decreased in advanced stages (H-Y III–V) [10, 23].

However, some studies have found no significant association between serum BDNF levels and the severity of motor symptoms, as assessed by the H-Y Scale [8, 12, 26–28], as well as by MDS-UPDRS part III [2, 24]. Notably, Rocha et al., the only study that also analyzed GDNF levels, likewise reported no significant associations [2]. All results are shown in Online Resource 5 (Suppl. Table 5).

### Neuropsychiatric Symptoms

Eighteen studies examined the relationship between BDNF, GDNF, and neuropsychiatric symptoms in Parkinson’s disease (PD), focusing on depression and cognitive decline. Detailed characteristics are presented in Online Resource 6 (Suppl. Table 6).

#### GDNF and BDNF Levels in Mood Disturbances

Seven studies evaluated BDNF and depression. Scalzo et al. and Siuda et al. did not find the association of depression and serum BDNF [6, 30]. Other authors assessed BDNF in CSF and serum, respectively, in PD patients with and without depression, including the effect of antidepressive treatment. Pålhagen et al. found no differences in CSF [31]. Ricci et al. found that PD-depressed patients presented lower BDNF serum levels compared with non-depressed PD patients [29]. Three studies analyzed the relation of serum BDNF with depression in PD patients without antidepressant and anxiolytic treatment [11, 12, 27]. The study of Wang et al. using the Zung Self-Rating Depression Scale (SDS) revealed that serum BDNF level was lower in the depressed PD group in comparison to the non-depressed PD group. SDS score and BDNF level were negatively correlated in both PD groups, and a multiple regression analysis showed that BDNF was an independent contributor to the SDS score in both PD groups [27]. Huang et al. utilized the Hamilton Depression Rating Scale 17 (HAMD-17) and presented consistent results [12]. The next study by Wang et al. also used HAMD-17, but with a different cut point for depression diagnosis, and found no differences [11]. Data for GDNF were limited. There was only one study, which observed no association of this NF with mood disorders [29].

In summary, three of seven studies revealed the association of a lower level of BDNF with depression in PD [12, 27, 29]. The studies also differ in the methods of depression diagnosis, including DSM-based diagnosis, self-rating scales, and clinician-administered scales [6, 11, 12, 27, 29–31]. The two studies assessed the relationship between antidepressant treatment and BDNF level in serum or CSF with opposite results [29, 31].

#### BDNF Levels in Cognitive Impairment

The relation of BDNF and cognitive function was addressed by seven heterogeneous studies, six evaluating serum BDNF, and one analyzing BDNF in CSF. Leverenz et al. found that CSF BDNF levels were related to cognitive performance in non-demented PD patients, but after adjusting for age, no significant associations remained with MMSE or other cognitive tests [35]. Costa et al. performed a comprehensive cognitive function assessment in PD-MCI and healthy controls, with MMSE > 25 pts for both groups, and found correlations of BDNF with executive functions and attention [33]. The study by Khalil et al. evaluated BDNF serum levels and cognitive performance measured with the Montreal Cognitive Assessment (MoCA) and revealed a significant relationship [34]. A similar association for some domains of MoCA was presented by Wang et al., with sex-dependent differences [11], and in another study evaluating this relation by Repeatable Battery for the Assessment of Neuropsychological Status (RBANS) [8]. Contrary results, that no association of BDNF levels was found, were exhibited in studies by Badr et al. and Ekemekyapar et al. [26, 32].

Four out of seven studies found a significant association of cognitive functions and BDNF in PD [8, 11, 33–35]. The studies had different inclusion criteria and used different tests. A few studies assessed particular cognitive function domains and most frequently, but not only, found an association with executive functions and attention problems [8, 11, 33, 35]. One of them suggested a difference related to gender, showing that lower BDNF levels were independently associated with better language performance in depressed males and better visuospatial/executive functioning in non-depressed females [11].

#### GDNF Levels in Cognitive Impairment

Five studies analyzed the relationship between GDNF serum levels and cognitive dysfunction in PD. Each of them exhibited that lower GDNF is associated with worse cognitive status [4, 13, 14, 36, 37]. The study conducted by Shi et al. also demonstrated that some GDNF precursors correlated with cognitive status and that GDNF was potentially valuable in the differential diagnosis of PD patients with cognitive impairment and normal cognition (AUC = 0.859) [14]. Tong et al. and Liu et al. identified associations between serum GDNF and executive function, language, attention, and working memory [13, 37]. A magnetic resonance imaging (MRI) study showed that GDNF was correlated with diffusion tensor image alterations in the internal capsule fibers linked to executive function impairment, and corpus callosum and cingulate gyrus—regions implicated in executive function, attention, and working memory impairments [4]. Tang et al., using functional MRI, found positive correlations between serum GDNF levels, MMSE, and MoCA scores and increased connectivity of the right inferior frontal gyrus, and negative correlations with decreased postcentral gyrus connectivity. A cortical thickness in the left frontal lobe and temporal lobes, especially the left caudal middle frontal lobe, was reduced in the low-GDNF PD group and associated with cognitive deficits [36].

### Other Non-motor Symptoms

Five studies have investigated the association between BDNF or GDNF and non-motor symptoms in PD other than neuropsychiatric symptoms. Of these, three focused on sleep disturbances [38–40], one on restless legs syndrome (RLS) [24], and one on constipation [41]. Each study examined individual non-motor symptoms, which severely limit the current understanding of the role of neurotrophins in the broader non-motor symptomatology of PD.

Wang et al. reported lower GDNF levels in PD patients with sleep disorders [38]. Similarly, Kaminska et al. found a moderate positive correlation between BDNF levels and the Epworth Sleepiness Scale (ESS); this association remained statistically significant after adjusting for confounding factors [40]. Jin et al. also found that PD patients with Rapid Eye Movement Sleep Behavior Disorders (RBD) presented lower serum BDNF, and it was an independent predictor of RBD [39]. Regarding RLS, only one study has explored BDNF levels in PD patients, revealing significantly lower BDNF concentrations in those with RLS and a strong negative correlation with the International Restless Legs Syndrome Study Group Rating Scale (IRLSSG-RS) [24].

In terms of gastrointestinal symptoms, just one study investigated the relationship between BDNF levels and constipation in PD. While BDNF levels were significantly lower in PD patients compared to controls, no significant difference was observed within the PD group based on the presence or absence of constipation [41]. All studies are characterized in the Online Resource 7 (Suppl. Table 7).

### Metabolic Effects

Four studies have examined BDNF levels concerning metabolic effects in Parkinson’s disease (PD), while none has investigated GDNF in this context. Among these, only the study by Alomari et al. reported a significant difference in lipid profiles between groups with low and high BDNF levels [43]. The remaining studies, which explored associations between BDNF and biomarkers such as electrolytes, C-reactive protein (CRP), lipopolysaccharides (LPS), creatinine, and vitamin D, found no significant associations [26, 42]. Additionally, another work by Alomari et al. suggested a potential role of BDNF in preserving vascular function in PD [44]. Detailed results are shown in the Online Resource (Suppl. Table 8).

### Confounding Factors

In the conducted systematic review, several potential confounding factors were identified that may have influenced the analyzed study outcomes. These factors are significant for understanding the limitations of the performed studies and explaining the observed inconsistency in the results concerning BDNF levels. All details are presented in the Online Resource 9 (Suppl. Table 9).

#### Dopaminergic Treatment

One of the key confounding variables identified is the influence of dopaminergic treatment on NF’s levels. Huang et al. evaluated interactions between BDNF and disease duration, motor impairment, and patient treatment in peripheral blood lymphocytes [10]. They found that patients treated with levodopa exhibited higher BDNF levels compared to untreated patients. In an earlier study, these authors revealed that serum BDNF was positively correlated with administered doses of levodopa [23]. In both studies, patients presented higher BDNF levels despite worse motor symptom assessment and longer disease duration [10, 23].

#### Neuroinflammation and Neurodegeneration

Another group of potential confounding factors identified relates to neuroinflammatory processes and neurodegenerative changes, which may influence BDNF levels independently of clinical severity or treatment. The relationships between BDNF levels and inflammation, as well as between BDNF levels and neurodegeneration, were explored in studies by Roy et al. and Ziebell et al., respectively [7, 45]. Roy et al. found that an increase in serum proinflammatory markers, such as IL-6 and TNF-alpha, was associated with a decrease in BDNF levels, but an increase in anti-inflammatory IL-10 was associated with higher BDNF [7]. Ziebell et al., using 123I-PE2I single photon emission computed tomography (SPECT), demonstrated that there was a significant correlation between serum BDNF levels and striatal neurodegeneration expressed as striatal [123I]PE2I striatal dopamine transporter binding [45].

#### Neuropsychiatric Treatment

Neuropsychiatric treatment is another factor that may influence BDNF levels. Ricci et al. reported that depressed PD patients treated with antidepressants presented higher BDNF serum levels than those without the medications [29]. Palhagen et al. found no statistical difference in BDNF assessed in CSF, before and after citalopram treatment [31]. Due to limited evidence, this factor requires further investigation, especially in the context of differences in biological sample types.

#### Variability of Biological Samples

There was notable variability of biological samples used across the analyzed studies. The vast majority of them measured serum BDNF levels [6–8, 11, 12, 22, 24–30, 32–34, 39, 40, 42, 45]. Alomari et al. conducted research in 2018 and 2022 using plasma samples [43, 44]. Some other authors evaluated blood cellular compartments. Chung et al. analyzed plasma exosomes [3], while Huang et al. assessed NF concentrations in peripheral blood lymphocytes [10]. Only two studies utilized CSF as the biological material [31, 35]. Although various biological materials were used, serum was the most common sample type among the included studies.

### Quality Assessment of Included Studies

The quality of the 35 included studies was evaluated using the Newcastle-Ottawa Scale (NOS) for case-control and cohort studies and an adapted version for cross-sectional studies. The detailed results of this assessment are presented in Online Resource 2.

The majority of studies were case-control studies (28/35), followed by cross-sectional studies (5/35) and cohort studies (2/35). Each study was assessed across eight domains (Q1–Q8), corresponding to key quality criteria specific to its design.

Overall, many studies demonstrated strengths in case definitions and the selection of cases and controls (Q1–Q2 for case-control studies), including Liu et al., Korkmaz et al., Tong et al., Jin et al., Wang et al., Roy et al., Shi et al., Ekmekyapar et al., Huang et al., Chung et al., Huang et al., Huang et al., Liu et al., Rocha et al., Wang et al., Khalil et al., Ventriglia et al., Ricci et al., Pålhagen et al., and Scalzo et al. [2–4, 6–8, 10, 12–14, 26, 28, 29, 31, 34, 37–39, 42]. However, several recurrent methodological limitations were evident, for example, the selection of controls (Q3) in case-control studies. The definition of controls (Q4) was insufficiently addressed in multiple studies, with some, including Korkmaz et al., Tang et al., Rocha et al., and Ricci et al., scoring zero stars for this domain [2, 29, 36, 42]. Non-response rates (Q8) were frequently marked as not applicable (n/a) or absent, as in the majority of case-control studies. Summarizing, 23/28 case-control studies reached 6* or more, which indicates good quality based on NOS (due to non-applicable Q8, the threshold was decreased from 7 to 6*).

The two presented cohort studies (Kaminska et al. and Yi et al.) [25, 40] generally scored positively in most domains, though some, such as Q4, Q7, and Q8, were marked n/a in Kaminska et al. (2022). According to NOS, the study by Yi et al. presented good quality (8*) [40].

Cross-sectional studies often satisfied representativeness (Q1; excluding Leverenz et al.) [35] and outcome assessment (Q6) criteria. However, all scored zero in sample size justification (Q2), and two (Ziebell et al. and Leverenz et al.) [35, 45] in the comparability of subjects (Q5). 4/5 cross-sectional studies (excluding the work by Leverenz et al.) [35] represented good quality according to NOS.

This quality assessment highlights methodological strengths and weaknesses within the evidence base that were considered when interpreting the systematic review’s findings.

## Discussion

### Critical Analysis of Findings

This review summarizes the 35 observational studies conducted between 2009 and 2025 that evaluated the connections of BDNF and GDNF with the clinical presentation of PD. Most of them analyzed BDNF concentration, and less frequently GDNF, in serum or plasma. Various methods were used, depending on the evaluated parameter. Although studies presented heterogeneous methodologies and endpoints, the main aim of the review was to consider the role of these NFs as a potential biomarker of the PD course, concerning both motor and non-motor aspects. Some of the presented results are inconsistent; however, significant relationships were identified that may provide valuable insight into the pathophysiology and course of the disease.

The relationship of motor symptoms and disease progression with BDNF is a main subject of interest in the reviewed papers. Only one research addressed GDNF in this context, reporting no significant associations [2]. Several studies have demonstrated that BDNF decreases with greater disease severity [3, 6, 7, 12], although Huang et al. (2021) revealed this reduction only in early stages [24]. Other authors reported contradictory findings [2, 8, 24, 26–28]. These inconsistencies may result from methodological heterogeneity and relatively small sample sizes across the studies. Different clinical assessment tools were applied, including the Hoehn-Yahr Scale [8, 26, 27], the UPDRS [3, 10, 24, 26], or the MDS-UPDRS [7, 22]. Scalzo et al. additionally employed functional tests, such as BBS or 6MWT [6]. Such methodological diversity likely contributed to the variability of findings.

Moreover, the majority of studies involved small cohorts, often comprising fewer than 100 participants, which limits statistical power. For instance, Ventriglia et al., who found no correlation between BDNF levels and the severity of symptoms, also reported significantly higher BDNF in PD patients compared to healthy controls [28], contradicting most previous studies [7–9]. The small sample size of 30 participants involved in this cohort could influence the reliability of these statistical findings.

Another possible cause of discrepancies in results may be variability in biological samples. Most studies used serum, whereas Huang et al. measured BDNF in peripheral blood lymphocytes, and Chung in plasma exosomes, which could have affected inter-study differences in results [3, 10].

Another inconsistency concerns the association between BDNF levels and disease duration. Di Lazzaro et al. reported that BDNF concentrations decrease with longer illness duration [22], whereas other studies have observed an increase in BDNF over time [6, 10, 24]. However, the correlation reported by Di Lazzaro et al. was weak (*r* = –0.187, *p* = 0.034), and no significant difference was found between patients with short (< 5 years) and long (> 5 years) disease duration. It appears inconsistent with the reported association [22]. The observed increase in BDNF levels with longer disease duration may be explained by the effects of pharmacotherapy. Similarly, this factor could contribute to inconsistencies in the results related to motor symptoms.

As it was mentioned, levodopa doses may elevate BDNF levels [10, 24]. There are several reports from preclinical studies confirming the impact of dopaminergic treatment on NFs levels [46, 47]. Zhang et al. demonstrated on rat PD models that repeated L-DOPA injections induced an increase of BDNF in the dopamine-depleted subthalamic nucleus [46], and other research showed GDNF elevation by dopamine agonists in cultured mouse astrocytes [47]. Moreover, associations have been suggested between BDNF and levodopa-induced dyskinesia [48]. Serum BDNF showed a positive correlation with motor severity and disease duration in levodopa-treated patients yet was also related to cognitive performance [23, 34]. This meant that in treated patients, BDNF became a marker of both medication exposure and adaptive neuroplasticity rather than a pure measure of neuroprotection. Therefore, the associations between BDNF and motor or cognitive performance had to be interpreted with caution, given the confounding influence of dopaminergic treatment.

Interesting data also comes from studies on neuropsychiatric symptoms. Some revealed lower BDNF serum levels in patients with depression [11, 12, 27, 29]; others found no such associations [6, 30, 31]. Several factors may contribute to these inconsistencies: different depression scales were used (e.g., HAM-D in most studies versus SDS in Wang et al. (2017)), and methodological differences such as measuring BDNF in CSF versus serum. Pålhagen et al. and Leverenz et al. evaluated BDNF concentrations in CSF [33, 35], whereas the other authors measured in serum [8, 27, 30, 33, 34]. Additionally, antidepressant treatment may affect results, as they are one of the probable confounding factors. Region-specific effects may help explain the seemingly contradictory findings. BDNF activity in the medial prefrontal cortex suppresses drug-seeking behavior and promotes antidepressant effects [49], whereas an equivalent BDNF increase in the ventral tegmental area and nucleus accumbens facilitates drug-seeking and enhances stress susceptibility [50]. Clinically, serum BDNF levels rise significantly only in patients who respond to or remit following antidepressant treatment [51]. Moreover, the proBDNF/mBDNF ratio (rather than absolute BDNF concentration) appears to be a more reliable biomarker, as it reflects the dynamic balance between opposing neuroplastic processes [52]. Within this mechanistic framework, it is therefore plausible that fluctuations in BDNF may lead to variable, and sometimes even opposing, outcomes in clinical and preclinical studies.

GDNF showed no significant associations specifically with mood disorders; however, it was assessed in only one study [29].

A similar methodological heterogeneity was noted in studies assessing cognitive associations with BDNF. While several studies confirmed the relationship [8, 11, 33–35], others found no association [26, 32].

Regarding GDNF and neuropsychiatric symptoms, research results are highly consistent despite methodological differences. All analyzed studies demonstrated a relationship between GDNF serum levels and cognition in PD [4, 13, 14, 34, 37]. Radiological imaging further supports these associations [36, 37]. These findings strongly suggest GDNF’s utility as a marker of cognitive dysfunction.

However, data on other disease aspects such as patients’ age, age at onset, non-motor symptoms associated with autonomic dysfunction, and metabolic effects remain very limited, based on single studies, making direct comparison difficult and thus requiring cautious interpretation [24, 26, 38, 40–42, 44, 53]. Most studies have significant limitations, including diverse study designs (case-control, cross-sectional, cohort) and varying methodologies, including various assessment tools and biological materials. Several were conducted by the same authors [8, 10, 12, 24, 27], which may contribute to selection bias. Additionally, the study populations were often poorly diversified, with many studies conducted in China [4, 8, 11, 13, 23–25, 27, 36–38, 41]. European and American populations were less examined. Another significant issue is the small number of patients in the study population, which was often less than 100 PD patients [2, 6, 10, 14, 23, 26, 28, 29, 31–36, 38, 40, 42, 44, 45].

Nevertheless, the reviewed evidence indicates that both BDNF and GDNF show potential as biomarkers of Parkinson’s disease, particularly GDNF in relation to cognitive impairment. Other aspects need to be deepened in well-designed further studies that address the limitations of previous research to better assess their actual clinical utility.

### Limitations

The authors aimed to prepare a comprehensive and objective overview of the current knowledge about BDNF and GDNF associations in Parkinson’s disease. This study has a number of limitations. First, the methodological heterogeneity of the included research, reflected in various study designs, assessment tools, and types of biological material, may influence the interpretation and formulation of conclusions. Furthermore, most studies on this topic focus on the Chinese population, which limits the ability to generalize the findings to other races and ethnic backgrounds. Some studies were conducted by overlapping research groups, which may have contributed to selection bias and could influence the interpretation of overall findings. The limited sample sizes of most studies disrupt statistical analysis, influencing the presentation of results as well. Other aspects that may affect the assessment include uncontrolled confounding variables, such as dopaminergic or neuropsychiatric treatment. Moreover, in some fields, the number of available studies remains insufficient to reliably conclude. These limitations should be considered during the interpretation of findings and highlight the need for further research.

### Future Directions

Based on the identified limitation, several directions for future research can be proposed. First, focusing on methodological standardization is essential, particularly concerning the selection of biological materials (serum, plasma, CSF, blood elements) and analytical methods used for BDNF and GDNF quantifications. Developing uniform protocols would enhance reproducibility between the studies.

The control of confounding variables should be incorporated. Further studies should include detailed documentation of dopaminergic and psychiatric medications, as these factors may alter NFs’ levels. It is also relevant to verify the actual impact of these factors.

Future research should involve larger, multicenter, and longitudinal studies to accurately evaluate the dynamics and connections of BDNF in relation to disease progression in motor and non-motor aspects.

There is also a need for more studies in Caucasian populations to determine the potential influence of ethnic backgrounds on neurotrophins’ levels.

Finally, more data regarding GDNF in the context of disease course is warranted. Promising and consistent associations with cognitive performance should be investigated for GDNF as a potential biomarker of cognitive decline or a potential target of neuroprotective interventions.

Proposed research priorities will be essential for validating the clinical utility of BDNF and GDNF and clarification of their role as biomarkers in Parkinson’s disease.

## Conclusions

BDNF may be associated with the clinical progression of PD, especially concerning motor symptoms, though results are conflicting. Non-motor symptoms of PD, especially mood disturbances and cognitive decline, are linked to NF levels. Findings regarding mood disturbances are also inconclusive. These discrepancies possibly result from differences in diagnostic instruments, biological sample types, dopaminergic treatment, and antidepressant use. Standardization of study protocols and harmonization of analytical methods are therefore essential to more clearly explain the inconsistent results and support translation into clinical practice.

Evidence on GDNF in the context of motor progression remains limited, while its association with cognitive impairment appears promising regarding its potential utility as a biomarker.

Future investigations should address these gaps to reach more reliable and conclusive findings. Substantial is accounted for by carefully designed studies and prioritizing multicenter, longitudinal approaches to establish reproducible evidence and guide biomarker-based therapeutic development.

## Supplementary Information

Below is the link to the electronic supplementary material.

ESM 1 (271 KB DOCX)

ESM 2 (29.8 KB DOCX)

ESM 3 (25.3 KB DOCX)

ESM 4 (16.4 KB DOCX)

ESM 5 (18.6 KB DOCX)

ESM 6 (22.8 KB DOCX)

ESM 7 (16.8 KB DOCX)

ESM 8 (15.9 KB DOCX)

ESM 9 (15.9 KB DOCX)

## Data Availability

Not applicable.
