# Supplementary material for: BDNF and GDNF in Parkinson’s Disease: Associations with Clinical Features, Disease Course, and Progression—A Systematic Review
Source: Mol Neurobiol. 2026 Feb 16;63(1):440. doi: 10.1007/s12035-025-05649-z (PMC12909441; doi:10.1007/s12035-025-05649-z)
Supplement: Supplementary file 2 — (29.8 KB DOCX) [file 12035_2025_5649_MOESM2_ESM.docx]

**BDNF and GDNF in Parkinson’s disease: associations with clinical features, disease course, and progression – a systematic review.**

**Authors:** Julia Węgrzynek-Gallina^1^, Aleksandra Buczek^2^, Jakub Malkiewicz^1^, Tomasz Chmiela^1^, Tomasz Gallina^3^, Patrycja Hudzińska^2^, Joanna Siuda^1^

^1^Department of Neurology, Faculty of Medical Sciences in Katowice, University Clinical Centre Prof K. Gibinski, Medical University of Silesia, 14 Medykow St. 40-752 Katowice, Poland.

^2^Students' Scientific Association, Department of Neurology, Faculty of Medical Sciences in Katowice, Medical University of Silesia, 14 Medykow St, 40-752 Katowice, Poland.

^3^Department of Cardiology and Structural Heart Disease, Faculty of Medical Sciences in Katowice, Medical University of Silesia, Upper-Silesian Medical Centre in Katowice, 45/47 Ziołowa St, Katowice, 40-635, Poland.

**Corresponding author:** Julia Węgrzynek-Gallina, [d201258@365.sum.edu.pl](mailto:d201258@365.sum.edu.pl)

| **Online Resource 2 - Detailed quality assessment of studies analyzed in the systematic review.**  The quality assessment was performed using the Newcastle–Ottawa Scale for the appropriate study types. | | | | | | | | | | |
| --- | --- | --- | --- | --- | --- | --- | --- | --- | --- | --- |
|  | **Referance** | **Study type** | **Q 1** | **Q 2** | **Q 3** | **Q 4** | **Q 5** | **Q 6** | **Q 7** | **Q 8** |
| 1 | Di Lazzaro et al. 2024 [22] | Cross-sectional-study | * | 0 | n/a | ** | ** | ** | ** |  |
| 2 | Wang et al. 2024 [11] | Cross-sectional study | * | 0 | n/a | ** | ** | ** | * |  |
| 3 | Liu et al. 2024 [4] | Case–control study | * | * | 0 | * | ** | * | * | n/a |
| 4 | Korkmaz et al. 2024 [42] | Case-control study | * | * | 0 | 0 | 0 | * | * | n/a |
| 5 | Tang et al. 2024 [36] | Case–control study | * | 0 | 0 | 0 | ** | * | * | n/a |
| 6 | Jin et al. 2023 [39] | Case-control study | * | * | 0 | 0 | ** | * | * | n/a |
| 7 | Badr et al. 2023 [32] | Case–control study | * | 0 | 0 | * | ** | * | * | n/a |
| 8 | Tong et al. 2023 [13] | Case-control study | * | * | * | * | ** | * | * | n/a |
| 9 | Wang et al. 2023 [27] | Case-control study | * | * | 0 | * | ** | 0 | * | n/a |
| 10 | Kaminska et al. 2022 [40] | Cohort study | * | * | * | n/a | * | * | n/a | n/a |
| 11 | Chen et al. 2022 [41] | Cross-sectional study | * | 0 | n/a | ** | ** | ** | * |  |
| 12 | Alomari et al. 2022 [43] | Case–control study | * | 0 | * | * | * | * | * | n/a |
| 13 | Roy et al. 2021 [7] | Case-control study | * | * | 0 | * | ** | * | * | n/a |
| 14 | Yi et al. 2021 [25] | Cohort study | * | * | * | * | * | * | * | * |
| 15 | Shi et al. 2021 [14] | Case–control study | * | * | 0 | * | * | * | * | n/a |
| 16 | Ekmekyapar et al. 2021 [26] | Case-control study | * | * | 0 | * | ** | * | * | 0 |
| 17 | Huang et al. 2021 [24] | Case-control study | * | * | * | * | ** | 0 | * | n/a |
| 18 | Huang et al. 2021 [12] | Case-control study | * | * | * | * | ** | * | * | n/a |
| 19 | Chung et al. 2020 [3] | Case-control study | * | * | 0 | * | ** | * | * | n/a |
| 20 | Huang et al. 2019 [10] | Case-control study | * | * | 0 | * | ** | * | * | n/a |
| 21 | Liu et al. 2020 [37] | Case-control study | * | * | 0 | * | * | * | * | n/a |
| 22 | Rocha et al. 2018 [2] | Case-control study | * | * | 0 | 0 | ** | * | * | n/a |
| 23 | Huang et al. 2018 [23] | Case-control study | * | 0 | 0 | * | * | * | * | n/a |
| 24 | Alomari et al. 2018 [44] | Case-control study | * | 0 | * | * | ** | * | * | n/a |
| 25 | Wang et al. 2017 [27] | Case-control study | * | * | * | * | ** | * | * | n/a |
| 26 | [Siuda et al. 2017](https://doi.org/10.1016/j.pjnns.2016.10.001) [30] | Case-control study | * | 0 | 0 | * | ** | * | * | n/a |
| 27 | Wang et al. 2016 [8] | Case-control study | * | * | * | * | ** | * | * | n/a |
| 28 | Costa et al. 2015 [33] | Case-control study | 0 | 0 | 0 | * | ** | * | * | n/a |
| 29 | Khalil et al. 2015 [34] | Case-control study | * | * | * | * | ** | * | * | n/a |
| 30 | Ventriglia et al. 2013 [28] | Case-control study | * | * | 0 | * | ** | * | * | n/a |
| 31 | Ziebell et al. 2012 [45] | Cross-sectional-study | * | 0 | n/a | ** | 0 | ** | * |  |
| 32 | Leverenz et al. 2011 [35] | Cross-sectional-study | 0 | 0 | n/a | ** | 0 | * | * |  |
| 33 | Ricci et al. 2010 [29] | Case-control study | * | * | 0 | 0 | * | * | * | n/a |
| 34 | Pålhagen et al. 2010 [31] | Case-control study | * | * | 0 | * | * | * | * | n/a |
| 35 | Scalzo et al. 2010 [6] | Case-control study | * | * | 0 | * | ** | * | * | n/a |

Table footnote: */** = criteria fulfilled (points awarded); 0 = criterion not fulfilled; n/a = not applicable.

Case-Control Studies: Q1 – Case definition adequate, Q2 – Representativeness of cases, Q3 – Selection of controls, Q4 – Definition of controls, Q5 – Comparability of cases and controls, Q6 – Ascertainment of exposure, Q7 – Same method of ascertainment for cases and controls, Q8 – Non-response rate.

Cohort Studies: Q1 – Representativeness of the exposed cohort, Q2 – Selection of the non-exposed cohort, Q3 – Ascertainment of exposure, Q4 – Demonstration that outcome of interest was not present at start of study, Q5 – Comparability of cohorts on the basis of design or analysis, Q6 – Assessment of outcome, Q7 – Was follow-up long enough for outcomes to occur, Q8 – Adequacy of follow-up of cohorts.

Cross-sectional study: Q1 – Representativeness of the sample, Q2 – Sample size, Q3 – Non-respondents, Q4 – Ascertainment of exposure (risk factor), Q5 – Comparability of subjects, Q6 – Assessment of the outcome, Q7 – Statistical test.
