# Supplementary material for: BDNF and GDNF in Parkinson’s Disease: Associations with Clinical Features, Disease Course, and Progression—A Systematic Review
Source: Mol Neurobiol. 2026 Feb 16;63(1):440. doi: 10.1007/s12035-025-05649-z (PMC12909441; doi:10.1007/s12035-025-05649-z)
Supplement: Supplementary file 3 — (25.3 KB DOCX) [file 12035_2025_5649_MOESM3_ESM.docx]

**BDNF and GDNF in Parkinson’s disease: associations with clinical features, disease course, and progression – a systematic review.**

**Authors:** Julia Węgrzynek-Gallina^1^, Aleksandra Buczek^2^, Jakub Malkiewicz^1^, Tomasz Chmiela^1^, Tomasz Gallina^3^, Patrycja Hudzińska^2^, Joanna Siuda^1^

^1^Department of Neurology, Faculty of Medical Sciences in Katowice, University Clinical Centre Prof K. Gibinski, Medical University of Silesia, 14 Medykow St. 40-752 Katowice, Poland.

^2^Students' Scientific Association, Department of Neurology, Faculty of Medical Sciences in Katowice, Medical University of Silesia, 14 Medykow St, 40-752 Katowice, Poland.

^3^Department of Cardiology and Structural Heart Disease, Faculty of Medical Sciences in Katowice, Medical University of Silesia, Upper-Silesian Medical Centre in Katowice, 45/47 Ziołowa St, Katowice, 40-635, Poland.

**Corresponding author:** Julia Węgrzynek-Gallina, [d201258@365.sum.edu.pl](mailto:d201258@365.sum.edu.pl)

**Online Resource 3. List of full-text articles excluded with reasons**

1. Holmuratova B., Rashidova N., Khalimova K., Khudayarova S. (2024). The role of brain-derived neurotrophic factor during Parkinson's disease, XXIX IAPRD World Congress on Parkinson's Disease and Related Disorders. Parkinsonism and Related Disorders.
   - wrong publication type (conference abstract)
2. Okhunova D., Rakhimbaeva G. (2024). The role of neurotrophic factors in development Parkinson's disease dementia, XXIX IAPRD World Congress on Parkinson's Disease and Related Disorders. Parkinsonism and Related Disorders.
   - wrong publication type (conference abstract)
3. Yilmaz M, Yay E, Balci N, et al. Parkinson's disease is positively associated with periodontal inflammation. J Periodontol. 2023;94(12):1425-1435.
   - wrong outcome
4. Matmurodov R., Muminov B., Abduqodirov E., Khalimova K. (2023). Glial neutrophic factor as a nonspecific factor in the progression of Parkinson's disease, 8th Asian and Oceanian Parkinson's Disease and Movement Disorders Congress, AOPMC 2023. Movement Disorders Clinical Practice.
   - wrong publication type (conference abstract)
5. Di Lazzaro G. (2022). NEURODEGENERATION AND INFLAMMATION IN PARKINSON'S DISEASE: AN INSIGHT FROM BLOOD BIOMARKERS, 52nd Annual Conference of the Italian Society of Neurology. Neurological Sciences.
   - wrong publication type (conference abstract)
6. Muminov B., Matmurodov R., Abduqodirov E. (2022). Changes in the level of glial neurotrophic factor in Parkinson's disease depending on the form, 2022 MDS International Congress. Movement Disorders.
   - wrong publication type (conference abstract)
7. Muminov B., Matmurodov R., Abduqodirov E. (2022). Level of glial neurotrophic factor in the blood plasma depending on the duration of Parkinson's disease, 2022 MDS International Congress. Movement Disorders.
   - wrong publication type (conference abstract)
8. Matmurodov M., Abduqodirov A., Khalimova L., Muminov B. (2022). Glial neurotrophic factor as an early diagnostic marker in Parkinson's disease, 2022 MDS International Congress. Movement Disorders.
   - wrong publication type (conference abstract)
9. Akbari, M., Gholipour, M., Hussen, B.M. et al. Expression of BDNF-Associated lncRNAs in Parkinson’s disease. Metab Brain Dis 37, 901–909 (2022).
   - wrong study design
10. Goger E., Yuksel G., Gursoy G. (2021). The effect of vitamin d in parkinson's disease, International Parkinson and Movement Disorder Society, MDS 2021. Movement Disorder. wrong publication type (conference abstract)
11. Alomari M., Khalil H., Khabour O., Alzoubi K. (2020). The importance of brain-derived neutrophic factor for lipid profile among Parkinson's patients, IAPRD XXV World Congress on Parkinson's Disease and Related Disorders, 2020. Parkinsonism and Related Disorders.
    - wrong publication type (conference abstract)
12. Roy A., Mondal B., Banerjee R., Choudhury S., Chatterjee K., Basu P., Dey S., Kumar H. (2020). Interplay of cytokines and nerve-growth factor in patients with Parkinson's Disease: A study in Eastern Indian population, IAPRD XXV World Congress on Parkinson's Disease and Related Disorders, 2020. Parkinsonism and Related Disorders.
    - wrong publication type (conference abstract)
13. Arslon D., Azizova D. (2020). Identified biomarkers for the development of cognitive impairment in Parkinson's disease, IAPRD XXV World Congress on Parkinson's Disease and Related Disorders, 2020. Parkinsonism and Related Disorders.
    - wrong publication type (conference abstract)
14. Delabary M., Monteiro E., Fagundes A., Casal M., Zanardi A.P., Boeno F., Munhoz S., De Menezes R., De Oliveira A., Martinez F., Tartaruga L.A., Haas A. (2020). Can clinical outputs predict BDNF levels in people with Parkinson's disease?, MDS International Congress. Movement Disorders.
    - wrong publication type (conference abstract)
15. Malczynska P., Brodacki B., Langfort J., Chalimoniuk M. (2018). Circulating levels of BDNF and microRNAs are associated with progression of idiopathic Parkinson's disease, 14th International Symposium "Molecular Basis of Pathology and Therapy in Neurological Disorders". Folia Neuropathologica.
    - wrong publication type (conference abstract)
16. Weintraub D. (2017). Multiple modality biomarker prediction of cognitive impairment in prospectively followed de novo Parkinson disease, 21st International Congress of Parkinsons Disease and Movement Disorders, MDS 2017. Movement Disorders.
    - wrong publication type (conference abstract)
17. Ho-Wo-Cheong D., O'Sullivan M., Mery V., Lafontaine A., Robinson A., Gros P., Martin J., Benedetti A., Kimoff R.J., Kaminska M. (2017). Inflammatory markers are associated with obstructive sleep apnea (OSA) in parkinson's disease (PD), American Thoracic Society International Conference, ATS 2017. American Journal of Respiratory and Critical Care Medicine.
    - wrong publication type (conference abstract)
18. De Pablos A.M., Garcia-Moreno J.M., Fernandez-Espejo E. (2016). Parkinsonian patients suffer from a serious disturbance of redox state in the central nervous system, as evaluated through the cerebrospinal fluid, 38th Congress of the Spanish Society of Physiological Sciences, SECF 2016. Journal of Physiology and Biochemistry.
    - wrong publication type (conference abstract)
19. Marti G., Saez N., Corominas M., Cuberas G., Lorenzo C., De Fabregues O., Alvarez-Sabin J., Casas M., Hernandez J. (2016). Nigrostriatal degeneration and serum BDNF levels in patients with “de novo” untreated Parkinson's disease, 20th International Congress of Parkinson's Disease and Movement Disorders. Movement Disorders.
    - wrong publication type (conference abstract)
20. Csencsits-Smith K., Suescun J., Gonzalez A., Actor A., Schiess M. (2016). Serum brain-derived neurotrophic factor as an indication of disease state in Parkinsonism and RBD, 20th International Congress of Parkinson's Disease and Movement Disorders. Movement Disorders.
    - wrong publication type (conference abstract)
21. Khalil H., Alomari M.A., Khabour O.F., Al-Hieshan A., Bajwa J.A. (2016). Circulatory levels of BDNF correlate with cognitive deficits in people with Parkinson's disease, 20th International Congress of Parkinson's Disease and Movement Disorders. Movement Disorders.
    - wrong publication type (conference abstract)
22. Gezen-Ak D., Bilgic¸ B., Hanaʇasi H., Ertan S., Lohmann E., Atasoy I.L., Alaylioʇlu M., Araz O.S., Önal B., Göundöuz A., Kiziltan G., Apaydin H., Gurvit H.I., Yilmazer S., Dursun E. (2015). Compromised regulation of serum cytokine levels and bdnf due to low levels of vitamin d in patients with early-or late-onsetalzheimer's disease or parkinson's disease, Alzheimer's Association International Conference 2015. Alzheimer's and Dementia.
    - wrong publication type (conference abstract)
23. Dursun E., Gezen-Ak D., Hanagasi H., Bilgic B., Lohmann E., Ertan S., Atasoy T.L., Alaylioglu M., Araz T.S., Onal B., Gunduz A., Apaydin H., Kiziltan G., Ulutin T., Gurvit H., Yilmazer S. (2015). The interleukin 1 alpha, interleukin 1 beta, interleukin 6 and alpha-2-macroglobulin serum levels in patients with early or late onset Alzheimer's disease, mild cognitive impairment or Parkinson's disease. Journal of Neuroimmunology, 50-57.
    - wrong population
24. Granholm L., Turner T., Boger H., Hinson V. (2013). Serum BDNF levels in patients with Parkinson's disease: Correlation with specific cognitive parameters, 17th International Congress of Parkinson's Disease and Movement Disorders. Movement Disorders.
    - wrong publication type (conference abstract)
25. Zanardini R., Ventriglia M., Bonomini C., Pasqualetti P., Sartori S., Lombardi F., Zanetti O., Volpe D., Gennarelli M., Bocchio Chiavetto L. (2012). Serum brain-derived neurotrophic factor levels are decreased in different dementias, 28th CINP World Congress of Neuropsychopharmacology. International Journal of Neuropsychopharmacology.
    - wrong publication type (conference abstract)
26. Ricci V., Pomponi M., Martinotti G., Bentivoglio A., Loria G., Caltagirone C., Bria P., Angelucci F. (2011). Antidepressant treatment restores brain-derived neurotrophic factor (BDNF) serum levels and ameliorates motor function in Parkinson's disease patients, 19th European Congress of Psychiatry, EPA 2011. European Psychiatry.
    - wrong publication type (conference abstract)
27. Palhagen S., Granerus A.K., Walinder J., Svenningsson P. (2010). Biomarker identification in CSF in patients with Parkinson's disease and major depression, 14th Congress of the European Federation of Neurological Societies, EFNS. European Journal of Neurology.
    - wrong publication type (conference abstract)
28. Leverenz J.B., Zabetian C., Peskind E.R., Watson G.S., Zhang J., Montine T.J. (2010). Cerebrospinal fluid biomarkers and cognitive performance in non-demented Parkinson's disease, 14th International Congress of Parkinson's Disease and Movement Disorders. Movement Disorders.
    - wrong publication type (conference abstract)
29. Teixeira, A. L., Barbosa, I. G., Diniz, B. S., & Kummer, A. (2010). Circulating levels of brain-derived neurotrophic factor: correlation with mood, cognition and motor function. Biomarkers in medicine, 4(6), 871–887.
    - wrong publication type (review)
30. Hinson V. K., Granholm A. C., Delambo A. , Boger H.A., Turner T. (2013). Serum EGF and BDNF levels in patients with parkinson's disease: correlations with PD executive dysfunction. 27th Annual Symposium on Etiology, Pathogenesis and Treatment. Movement Disorders 28(10):E9
    - wrong publication type (conference abstract)
31. Salehi Z, Mashayekhi F. Brain-derived neurotrophic factor concentrations in the cerebrospinal fluid of patients with Parkinson's disease. J Clin Neurosci. 2009;16(1):90-93.
    - wrong outcome
32. Sadanand, A., Janardhanan, A., Vanisree, A. J., & Pavai, T. (2018). Neurotrophin Expression in Lymphocytes: a Powerful Indicator of Degeneration in Parkinson's Disease, Amyotrophic Lateral Sclerosis and Ataxia. *Journal of molecular neuroscience : MN*, *64*(2), 224–232.
    - wrong population
33. Przybylska, I., Marusiak, J., Toczyłowska, B., Stępień, A., Brodacki, B., Langfort, J., & Chalimoniuk, M. (2024). Association between the Val66Met (rs6265) polymorphism of the brain-derived neurotrophic factor (BDNF) gene, BDNF protein level in the blood and the risk of developing early‑onset Parkinson's disease. *Acta neurobiologiae experimentalis*, *84*(3), 296–308.
    - wrong study outcome
