# Supplementary material for: BDNF and GDNF in Parkinson’s Disease: Associations with Clinical Features, Disease Course, and Progression—A Systematic Review
Source: Mol Neurobiol. 2026 Feb 16;63(1):440. doi: 10.1007/s12035-025-05649-z (PMC12909441; doi:10.1007/s12035-025-05649-z)
Supplement: Supplementary file 4 — (16.4 KB DOCX) [file 12035_2025_5649_MOESM4_ESM.docx]

Online Resource 4 (Suppl. Table 4.) Overview of studies assessing the associations between BDNF and GDNF levels and age of onset and disease duration in Parkinson’s disease.

| **Number** | **Reference** | **Neurotrophin** | **Study groups: n** | **Summary of results** |
| --- | --- | --- | --- | --- |
| 1 | Di Lazzaro et al. 2024[22] | BDNF | PD-short disease duration: 35  PD-long disease duration: 69 | BDNF level negatively correlated with:  - age (r= -0.245, p=0.014)  - disease duration (r= -0.187, p=0.034)  There is no statistical difference between short (<5 years) and long (>5 years) disease duration. |
| 2 | Huang et al. 2021[24] | BDNF | PD with RLS: 53  PD without RLS: 196 | There was no significant correlation between BDNF and the disease duration and age of onset. |
| 3 | Yi et al. 2021 [25] | mBDNF, proBDNF | ex-PD: group 111  ex-NPD: group 45 | At baseline serum proBDNF levels were significantly higher in the ex-PD (with PD) group than in the ex-NPD (without PD)group (235.49±60.75 vs.191.75±66.12 ng/ml, p=0.0001), while  mBDNF and mBDNF/proBDNFratio were significantly lower in the ex-PD group than in the ex-NPD group (mBDNF: 19.73±7.31 vs. 23.47±8.21 ng/ml, p=0.0059; mBDNF/proBDNF: 0.09±0.05 vs. 0.15±0.12, p<0.0001).  At 1-year follow-up, serum levels of mBDNF and mBDNF/proBDNF in the po-PD (with PD) group were significantly lower than in the po-NPD (without PD) group (mBDNF: 19.24±7.20 vs. 25.05±7.67 ng/ml, p<0.0001; mBDNF/proBDNF 0.09±0.05 vs. 0.16±0.14, p<0.0001).  Serum proBDNF levels were significantly higher in the po-PD group than in the po-NPD group (235.56±60.80 vs.188.42±65.08 ng/ml, p<0.0001).  In ROC curve analysis, both at baseline and 1-year follow-up, the mBDNF/proBDNF ratio has better diagnostic value than mBDNF or proBDNF alone in the  diagnosis of early PD. |
| 4 | Huang et al. 2019 [10] | BDNF | PD: 28 | BDNF level in peripheral blood lymphocytes positively correlated with disease duration (r =0.644, p<0.001). |
| 5 | Rocha et al. 2018 [2] | BDNF, pro-BDNF, GDNF | PD: 40 | The neurotrophic factors were not associated with disease duration. |
| 6 | Huang et al. 2018 [23] | BDNF | PD: 60 | BDNF decreased with the later age of onset (r= -0.385, p=0.002).  BDNF level increased with the longer disease duration  (r =0.879, p<0.001). |
| 7 | Scalzo et al. 2010 [6] | BDNF | PD: 47 | BDNF correlated positively with disease duration (r= 0.526, p<0.001). |

**Abbreviations:** PD - Parkinson’s disease, RLS - Restless legs syndrome, BDNF - Brain-derived neurotrophic factor, GDNF - Glial-derived neurotrophic factor
