# Supplementary material for: BDNF and GDNF in Parkinson’s Disease: Associations with Clinical Features, Disease Course, and Progression—A Systematic Review
Source: Mol Neurobiol. 2026 Feb 16;63(1):440. doi: 10.1007/s12035-025-05649-z (PMC12909441; doi:10.1007/s12035-025-05649-z)
Supplement: Supplementary file 5 — (18.6 KB DOCX) [file 12035_2025_5649_MOESM5_ESM.docx]

Online Resource 5 (Suppl. Table 5) Overview of studies assessing the associations between BDNF and GDNF levels and motor severity in Parkinson’s disease.

| **Number** | **Reference** | **Neurotrophin** | **Study groups: n** | **Assessment tools** | **Summary of results** |
| --- | --- | --- | --- | --- | --- |
| 1 | Di Lazzaro et al. 2024 [22] | BDNF | PD-short disease duration: 35  PD-long disease duration: 69 | MDS-UPDRS parts II-IV, H-Y Scale | Tremor-dominant patients showed higher BDNF levels (p=0.045). |
| 2 | Roy et al. 2021 [7] | BDNF | PD: 27 | MDS-UPDRS part III, H-Y Scale | BDNF decreased with the increase of motor impairment (MDS-UPDRS part III (r= -0.823, p=0.001).  BDNF levels significantly decreased with PD severity (H-Y I vs H-Y III, p=0.0001). |
| 3 | Huang et al. 2021 [24] | BDNF | PD with RLS: 53  PD without RLS: 196 | UPDRS total score, H-Y Scale | There was no significant correlation between BDNF and the H-Y stage, UPDRS score in PD with RLS, and without it. |
| 4 | Ekmekyapar et al. 2021 [26] | BDNF | PD-MCI: 36  PD with mild dementia: 19  PD with moderate dementia: 8 | UPDRS parts I-IV and total score, H-Y Scale | There was no significant relationship between BDNF serum levels and H-Y scale. |
| 5 | Huang et al. 2021 [12] | BDNF | PD with depression: 122  PD without depression: 137 | UPDRS part III, H-Y Scale | BDNF was negatively correlated with severity of PD assessed by the UPDRS part III and H-Y scale (r= -0.541,p<0.001 and r= - 0.448, p<0.001 respectivly). |
| 6 | Chung et al. 2020 [3] | BDNF | PD: 114 | UPDRS part III, H-Y Scale | Lower plasma exosomal BDNF levels correlated with more severe axial motor symptoms, such as postural instability (p=0.003), arising from the chair (p=0.036) and gait impairment (p=0.014) assessed in UPDRS part I-III. |
| 7 | Huang et al. 2019 [10] | BDNF | PD: 28 | UPDRS total score,  H-Y Scale | BDNF level in peripheral blood lymphocytes positively correlated with the severity of motor impairment (UPDRS: r=0.754, p<0.001 and r=0.757, p<0.001 in H-Y). |
| 8 | Rocha et al. 2018 [2] | BDNF, pro-BDNF, GDNF | PD: 40 | UPDRS I-III, H-Y Scale | The neurotrophic factors levels were not associated with the degree of motor or functional impairment (UPDRS part I-III).  There was no significant difference between PD patients and controls regarding the plasma levels of the evaluated neurotrophic factors (BDNF, pro-BDNF, GDNF, and others). |
| 9 | Huang et al. 2018 [23] | BDNF | PD: 60 | UPDRS total score,  H-Y Scale | BDNF level in early stages (H-Y I-II) is significantly lower compared to mean BDNF in healthy controls (1924.83 ±331.24 vs. 4649.84 ±315.86 pg/ml, p<0.001).  BDNF level in more advanced stages (H-Y III and IV-V) is significantly higher than in healthy controls (consecutively 8000.84 ±389.83 vs. 4649.84 ±315.86pg/ml, p=0.001 and 8644.65 ±382.08c vs. 4649.84 ±315.86pg/ml, p<0.001).  BDNF concentration in the early stages is reduced.  BDNF level increased with the increase of:  - UPDRS part I-III (r=0.874, p<0.001)  - H-Y stage (r=0.907, p<0.001) |
| 10 | Wang et al. 2017 [27] | BDNF | PD with depression: 46  PD without depression: 50 | H-Y scale | There was no significant correlation between BDNF serum levels and H-Y scale. |
| 11 | Wang et al. 2016 [8] | BDNF | PD: 97 | H-Y scale | There was no significant correlation between BDNF serum levels and H-Y scale. |
| 12 | Ventriglia et al. 2013 [28] | BDNF | PD: 30 | H-Y scale | There was no correlation between BDNF level and severity of symptoms measured on the H-Y scale (p=0.45). |
| 13 | Scalzo et al. 2010 [6] | BDNF | PD: 47 | UPDRS I-III and total score, H-Y scale, BBS, TUG, 6MWT, CGS | BDNF correlated with:  -motor impairment assessed in UPDRS part II-III (r=0.458, p<0.001 and r=0.439, p=0.002) and severity symptoms measured in H-Y (r=0.355, p=0.014)  - poor balance on the BBS (r= -0.379, p=0.009), as well as with more time spent at the TUG (r= 0.430, p=0.003), reduced gait speed in CGS(r= -0.450, p=0.001), and shorter distance covered in the 6MWT (r= -0.488, p=0.001). |

**Abbreviations:** PD – Parkinson’s disease, PD-MCI – Parkinson’s disease mild cognitive impairment, RLS – Restless legs syndrome, BDNF – Brain-derived neurotrophic factor, GDNF – Glial-derived neurotrophic factor, UPDRS – Unified Parkinson’s Disease Rating Scale, MDS-UPDRS – Movement Disorder Society-sponsored revision of the Unified Parkinson’s Disease Rating Scale, H–Y scale – Hoehn and Yahr scale, BBS – Berg Balance Scale, TUG – Timed Up and Go test, 6MWT – 6-Minute Walk Test, CGS – Comfortable Gait Speed
