# Supplementary material for: BDNF and GDNF in Parkinson’s Disease: Associations with Clinical Features, Disease Course, and Progression—A Systematic Review
Source: Mol Neurobiol. 2026 Feb 16;63(1):440. doi: 10.1007/s12035-025-05649-z (PMC12909441; doi:10.1007/s12035-025-05649-z)
Supplement: Supplementary file 6 — (22.8 KB DOCX) [file 12035_2025_5649_MOESM6_ESM.docx]

Online Resource 6 (Suppl. Table 6) Overview of studies assessing the associations between BDNF and GDNF levels and neuropsychiatric symptoms in Parkinson’s disease.

| **Number** | **Reference, year** | **Neurotrophin** | **Study groups: n** | **Assessment tools** | **Summary of results** |
| --- | --- | --- | --- | --- | --- |
| 1 | Wang et al. 2024 [11] | BDNF | PD with depression: 108  PD without depression: 108 | MoCA, HAM-D | In male PD patients with depression, serum BDNF levels were negatively correlated with the language factor (r = –0.424, p = 0.013; Beta = –0.424, t = –2.647, p = 0.012) In female PD patients without depression, BDNF levels were negatively correlated with the visuospatial/executive factor (r = –0.407, p = 0.011) and independently associated with both MoCA total score (Beta = –0.293, t = –2.102, p = 0.043) and the visuospatial/executive index (Beta = –0.482, t = –3.865, p < 0.001) |
| 2 | Liu et al. 2024 [4] | GDNF | PD-N: 52  PD-MCI: 53 | MMSE, MoCA, DSB, TMT-A and B, CDT, BNT, VFT, CCT, AVLT-H, MRI with DTI and VBA | The serum GDNF level was significantly lower in PD-MCI compared to PD-N and healthy controls (p<0.05).  In the PD-MCI group, there was a significant correlation with GDNF levels in the following regions in MRI:  -left internal capsule (r=0.342, p=0.025), which was additionally clinically correlated with TMT-B and CDT (evaluated executive functions)  -right cingulate gyrus (r=0.655, p=0.001), which was additionally associated with AVLT-H (assessing memory function)  -right corpus callosum (r=0.407,p = 0.018) and left corticospinal tract (r=0.528, p=0.006).  There was no significant correlation with other abnormal white matter fibers.  The serum levels of GDNF in the healthy controls and PD-N groups did not show a significant correlation with abnormal white matter fibers. |
| 3 | Tang et al. [36] | GDNF | PD-high-GDNF: 19  PD-low-GDNF: 19 | MoCA, MMSE, rs-fMRI | PD-high-GDNF presented significantly better MoCa and MMSE score than PD-low-GDNF (19.95± 4.552 vs. 17.21± 3.750, p=0.013 and 24.26± 3.280 vs. 21.21± 3.441, p=0.001).  It was observed that the higher the serum GDNF in PD, the higher connectivity of cluster1 (right cerebrum Frontal lobe Inferior Frontal Gyrus) and the better the cognition status.  It was observed the higher the serum GDNF of PD patients, the lower the cluster2 (right cerebrum Parietal lobe Postcentral Gyrus) connectivity and the better the cognitive evaluation.  The combined detection can improve the accuracy of screening for the cognitive impairment of PD patients. The AUC value was 0.956(95%CI: 0.906, 1.00, p= 0.000) after cluster1, cluster2, and serum GDNF markers were combined.  The following combination of indicators could differentiate the PD group from the mild and moderate cognition impairment groups based on MMSE:  the AUC of combination indicator 2 (DC of clusters 1 and 2, serum GDNF, duration, LEDD) was 0.808 (95% CI: 0.581, 1.00, p=0.032, Youden's index = 56%).  Based on MoCa, the optimal diagnostic biomarker combination (DC of clusters 1 and 2, serum GDNF, duration, LEDD) proved to be the most accurate combination assay for the detection of cognitive status (AUC = 0.795, 95%CI: 0.601, 0.990, p=0.015, Youden's index = 52.3%). |
| 4 | Badr et al. [32] | BDNF | PD:58 | MoCA, Mattis CDRS, PD–CRS, SCOPA–COG, HAM-D, BDI-II, MRI with DTI and SWI | Serum BDNF presented no statistically significant correlations with the conducted neuropsychiatric assessment. |
| 5 | Tong et al. 2023 [13] | GDNF | PD-N: 44  PD-MCI: 41  PD-D: 20 | DSF, DSB, Total digit span, TMT-A, TMT-B, CDT, SFT, BNT, AVLT-H free recall, CCT | The serum GDNF concentration of the PD-N group was significantly higher than that of the PD-MCI group (532.13 ± 138.30 pg/mL vs. 439.87 ± 139.59 pg/mL, p<0.01) and the PD-D group (532.13±138.30 pg/mL vs. 424.73 ± 101.96 pg/mL, p<0.01).  There was no statistically significant difference in the serum GDNF level between the PD-MCI group and the PD-D group (p=0.899).  GDNF serum in PD correlated negatively with executive function assessment conducted with TMT-A and TMT-B scores (r = -0.329, p=0.001, and r= -0.304, p=0.002) and positively correlated with SFT (r=0.275, p<0.05).  GDNF was correlated positively with AVLT-H assessing memory (r=0.252, p<0.05).  There was no correlation with the HC groups. |
| 6 | Shi et al. 2021 [14] | GDNF, α-pro-GDNF, β-pro-GDNF | PD-N: 26  PD-CI: 27 | MMSE, MoCA, CDR | GDNF level in the PD-N group was significantly higher than the PD-CI group (679.43±175.58 vs. 444.15±96.11, p<0.001). There was no significant difference between the healthy control and PD-CI groups.  There were positive correlations between GDNF levels and the MMSE and MoCA scores (r=0.610, p<0.001 and r=0.579, p< 0.001, respectively), and a negative correlation between GDNF levels and CDR scores (r=-0.573, p< 0.001). The regression analysis confirmed that GDNF level had a significant effect on cognition and was a variable affecting MMSE, MoCA, and CDR score.  ROC curve analysis revealed the diagnostic accuracy was as follows: AUC = 0.859, P < 0.001, 95% confidence interval: 0.736–0.939, with the best cut-off value of serum GDNF levels for PD-CI diagnosis was 508.991 pg/mL (sensitivity of 85.19%, and specificity of 84.62%). Diagnostic accuracy of the combination of GDNF and its precursors (GDNF/α-pro-GDNF ratio,  and GDNF/β-pro-GDNF) was not better than GDNF alone. |
| 7 | Ekmekyapar et al. 2021 [26] | BDNF | PD-MCI: 36  PD with mild dementia: 19  PD with moderate dementia: 8 | WMS, MMSE | There was no significant relationship between BDNF serum levels and cognitive levels of the patients. |
| 8 | Huang et al. 2021 [12] | BDNF | PD with depression: 122  PD without depression: 137 | HAM-D | BDNF levels in PD with depression were significantly lower than in those without depression (p<0.001).  The serum BDNF cutoff value of 3646 pg/ml had an 80% sensitivity and a 95% specificity for distinguishing between PD patients with depression and without depression, with an area under the curve of 0.91.  In depressed PD, the HAMD-17 score was negatively correlated with BDNF levels (the higher HAMD-17, the lower BDNF; r= -0.59, p<0.001) and positively correlated with UPDRS part III score and H-Y stage (r=0.51, p<001). Multiple regression analysis showed that BDNF levels and UPDRS III score were independent contributors to the HAMD-17 score in depressed PD patients.  In PD without depression, BDNF levels, H-Y stage, and UPDRS part III score were not associated with HAMD-17. |
| 9 | Liu et al. 2020 [37] | GDNF | PD-N: 44  PD-MCI: 41  PD-D: 20 | MMSE, MoCA, TMT-A, TMT-B, CDT, BNT, AVLT, CCT, Digit span backward Test, VFT - Verbal Fluency Test, GDS‐15 | Among three groups of PD patients, the more serious cognitive dysfunction, the lower level of serum GDNF (PD-N vs. PD-MCI: 455.56 pg/ml vs. 384.44 pg/ml, p<0.05; PD-MCI vs. PD-D: 384.44 pg/ml vs. 280.46 pg/ml, p<0.05.  GDNF showed some correlation in PD-MCI group:  - it was positively related to Digital Span (back word) Test (r=0.388, p<0.05), BNT (r=0.407, p<0.01), and Semantic fluency (animals) Test (r=0.350, p<0.05), assessing attention and working memory,  - it was negatively correlated with TMT-A and TMT-B (r= -0.463, p<0.01 and r= -0.371, p<0.05), assessing executive function. |
| 10 | Siuda et al. 2017 [30] | BDNF | PD: 49 | MMSE, CDT, AVLT, TMT-A, TMT-B, BDI | There is no association between serum BDNF and depression. |
| 11 | Wang et al. 2017 [27] | BDNF | PD with depression: 46  PD without depression: 50 | SDS | BDNF serum levels were significantly lower in patients with depression than in those without depression (4.96 ±1.34 vs. 9.61 ± 1.66 ng/ml, p < 0.001 and after covarying for age, sex, BMI, education, smoking status, and H-Y, this significant difference still existed.  Both in depressed and non-depressed PD patients, BDNF was negatively correlated with mood assessment by SDS score (r= - 0.54, p < 0.001 and r= -0.44, p < 0.001, respectively). Multiple regression analysis showed BDNF was an independent contributor to the SDS score in both groups. |
| 12 | Wang et al. 2016 [8] | BDNF | PD: 97 | RBANS | The correlation analysis showed a significant positive association between BDNF and the RBANS total score and all five indexes, including attention (r= 0.73), delayed memory (r= 0.60, p<0.001), language (r= 0.58, p<0.001), visuospatial/constructional (r=0.58, p<0.001) and immediate memory (r= 0.57, p<0.001). Multiple regression showed BDNF was an independent contributor to the RBANS total score and all parts. |
| 13 | Costa et al. 2015 [33]. | BDNF | PD: 13 | MMSE, Immediate and Delayed Recall of a 15-Word List, Prose Recall, Immediate and Delayed reproduction of Rey’s Figure, DST, CBTT, TMT-A, VFT, MCST, RCPM, TMT-B, Objects and Verbs Naming subtests from the Neuropsychological Examination of Aphasia, Copy of Drawings and Copy of Drawings with Landmarks, Copy of Rey’s Figure, Zoo Map test, Stroop test, BDI | There was a significant positive correlation between BDNF serum levels and executive (r=0.62, p=0.023) and attention domains (r=0.59, p=0.032). Other domains did not gain statistical significance.  In analysis involving the different components of the executive domain there was found a positive correlation BDNF and self-monitoring/response inhibition (r=0.68, p=0.011) and working memory (r=0.62, p=0.025). |
| 14 | Khalil et al. 2016 [34] | BDNF | PD: 29 | MoCA | BDNF level correlated positively with MOCA total score (r=0.44, p<0.012). In linear regression analysis, it was found that BDNF serum significantly predicts cognitive function score, accounting 19% of the variance of the MOCA total score (MOCA = 12.9 + 0.41BDNF; R2 = 0.19; p=0.016). |
| 15 | Leverenz et al. 2011 [35] | BDNF | PD: 22 | MMSE, Logical Memory II, Category Fluency (animal& vegetable), Digit Symbol, TMT-A, TMT-B | CSF BDNF concentration was significantly correlated positively with Digit Symbol performance (r= 0.54, p=0.01), but with no other neuropsychological test result. The association was not quite significant after adjusting for age (p=0.06). |
| 16 | Scalzo et al. 2010 [6] | BDNF | PD with depression: 14  PD without depression: 33 | BDI, MMSE | BDNF levels did not correlate with BDI scores. Furthermore, there was no difference in serum BDNF levels between PD patients with, and without depression (p = 0.762). BDNF did not correlate significantly with MMSE. |
| 17 | Pålhagen et al. 2010 [31] | BDNF | PD without depression: 14  PD with major depression: 11 | MADRS, HAM-D, | There was no statistically significant difference in BDNF serum level between PD and PD with major depression or other-only major depression group at baseline. Furthermore, there was no statistical difference in BDNF before and after citalopram treatment in PD and PD with major depression. After the treatment, BDNF was lower in PD with major depression than in the major depression group without PD. |
| 18 | Ricci et al. 2010 [29] | BDNF, GDNF | PD with depression: 26  PD without depression: 20 | HAM-D | PD-depressed patients presented lower BDNF serum levels compared with nondepressed PD patients (p<0.01).  No significant difference in GDNF serum levels was observed. |

**Abbreviations:** PD - Parkinson’s disease, PD-D - Parkinson's disease with dementia, PD-MCI - Parkinson's disease with mild cognitive impairment, PD-N - Parkinson's disease with normal cognitive function, BDNF - Brain derived neurotrophic factor, GDNF - Glial derived neurotrophic factor, PD-high-GDNF - Parkinson's disease with high serum GDNF, PD-low-GDNF - Parkinson's disease with low serum GDNF, HAM-D - Hamilton Rating Scale for Depression, MoCA - The Montreal Cognitive Assessment, DSB - Digit Span Backward, TMT-A - Trail Making Test Part A, TMT-B - Trail Making Test Part B, AVLT-H free recall - Auditory Verbal Learning Test - H, VFT- Verbal fluency test, BDI - Beck Depression Inventory, BNT - Boston Naming Test, CCT - Color-Cued Task, MMSE - Mini-Mental State Examination, MRI with DTI and VBA - Magnetic Resonance Imaging with Diffusion Tensor Imaging and Voxel-Based Analysis, rs-fMRI - Resting- state functional Magnetic Resonance Imaging, CDRS - Clinical Dementia Rating Scale, PD-CRS - The Parkinson's Disease-Cognitive Rating Scale, SCOPA–COG - Scales for Outcomes of Parkinson's disease-Cognition, MRI with DTI and SWI - Magnetic Resonance Imaging with Diffusion Tensor Imaging and Susceptibility-Weighted Imaging, CDT - Clock Drawing Test, SFT - Semantic Fluency Test, BNT - Boston Naming Test, VFT - Verbal Fluency Test, GDS-15 - Geriatric Depression Scale-15, WMS - Wechsler Memory Scale, SDS - Sheehan Disability Scale, RBANS - Repeatable Battery for the Assessment of Neuropsychological Status, DST - Digit Span Test, CBTT - Corsi Block Tapping Test, MCST - Maintenance Cognitive Stimulation Therapy, RCPM- Raven’s Coloured Progressive Matrices, MADRS - The Montgomery–Åsberg Depression Rating Scale
