# Supplementary material for: BDNF and GDNF in Parkinson’s Disease: Associations with Clinical Features, Disease Course, and Progression—A Systematic Review
Source: Mol Neurobiol. 2026 Feb 16;63(1):440. doi: 10.1007/s12035-025-05649-z (PMC12909441; doi:10.1007/s12035-025-05649-z)
Supplement: Supplementary file 7 — (16.8 KB DOCX) [file 12035_2025_5649_MOESM7_ESM.docx]

Online Resource 7 (Suppl. Table 7) Overview of studies assessing the associations between BDNF and GDNF levels and non-motor symptoms linked to autonomic dysfunction in Parkinson’s disease.

| **Number** | **Reference, year** | **Neurotrophin** | **Study groups: n** | **Non-motor symptoms assessed** | **Non-motor assessment tools** | **Summary of results** |
| --- | --- | --- | --- | --- | --- | --- |
| 1 | Wang et al. 2023 [38] | GDNF | PD-NSD: 36  PD-SD: 51 | Sleep disturbances | RBD-SQ, ESS, PSQI, PDSS, NMSS | PD with sleep disturbances presented lower serum GDNF levels than those without sleep disturbances (287.55 ± 85.92 vs. 392.81 ± 85.08, p<0.001). |
| 2 | Jin et al. 2023 [39] | BDNF | PD-nRBD: 56  PD-RBD: 45 | Sleep disturbances | RBDQ-HKRBDSQ | PD with RBD presented lower serum BDNF levels compared to PD without RBD (32.86 ng/ml vs. 42.58 ng/ml, p<0.001). BDNF levels were identified as an independent predictor of RBD. |
| 3 | Kaminska et al. 2022 [40] | BDNF | PD without OSA: 20  PD with OSA and CPAP: 22  PD with OSA and without CPAP: 24 | Sleep disturbances | polismonography, ESS, MDS-UPDRS part I | There was a non-significant association between BDNF and sleep time, and inversely with time awake after sleep onset. A six-month change in BDNF showed a trend toward association with changes in ESS (r=0.33, p=0.049). Adjusting for age, sex, and BMI, change in BDNF was positively associated with the change in ESS (beta= 1450, 95% CI 244-2655, p=0.02). |
| 4 | Chen et al. 2022 [41] | GDNF | Cons-Pro-PD: 48  nCons-PD: 49  Cons-clinic-PD: 31 | Constipation | NMSS, MDS-UPDRS-I scores, ROME IV functional constipation criteria, PAC-QOL, PAC-SYM | PD without constipation presented higher GDNF levels than the prodromal stage constipation group (528.44 pg/ml vs.360.72 pg/ml, p=0.000) and the clinical stage constipation group (528.44 pg/ml vs. 331.36 pg/ml, p=0.000), with no statistically significant differences found between the prodromal and clinical constipation groups. |
| 5 | Huang et al. 2021 [24] | BDNF | PD with RLS: 53  PD without RLS: 196 | RLS | IRLSSG-RS | BDNF serum levels were significantly lower in PD patients with RLS than those without RLS (2604.40 ± 1011.22 pg/ml vs. 3814.57 ± 835.38 pg/ml, p<0.001) and in healthy controls. After adjusting for sex, age, education, BMI, alcohol, and smoking status, this difference was still significant. It was correlated negatively with IRLSSG-RS score (r=−0.639, p<0.001). After multivariate regression analysis, the correlation was still statistically significant.  In ROC analysis BDNF cutoff value of 3,638 pg/ml had a 78% sensitivity and an 81% specificity for distinguishing between PD patients with RLS and without RLS, with an area under the curve of 0.82 |

**Abbreviations:** PD - Parkinson’s disease, BDNF - Brain-derived neurotrophic factor, GDNF - Glial-derived neurotrophic factor, PD-SD - PD with sleep disturbance, PD-NSD - PD without sleep disturbance, Cons-clinic-PD - Parkinson's disease with clinical stage constipation, Cons-Pro-PD - Parkinson's disease with prodromal stage constipation, nCons-PD - Parkinson's disease without constipation, PD with OSA and CPAP - Parkinson's disease with obstructive sleep apnea who are using Continuous Positive Airway Pressure treatment, PD with OSA without CPAP- Parkinson's disease with obstructive sleep apnea who are not using Continuous Positive Airway Pressure treatment, PD with RLS - Parkinson's Disease with Restless Legs Syndrome, PD without RLS - Parkinson's Disease without Restless Legs Syndrome, RBD-SQ-REM - Sleep Behavior Disorder Screening Questionnaire, ESS - Epworth Sleepiness Scale, PSQI - Pittsburgh Sleep Quality Index, PDSS - Parkinson's Disease Sleep Scale, NMSS - Non-motor Symptom Scale, MDS-UPDRS - Movement Disorder Society sponsored revision of the Unified Parkinson's Disease Rating Scale, NMSS - The Non-Motor Symptoms Scale, PAC-QOL - Patient Assessment of Constipation Quality of Life, PAC-SYM - Patient Assessment of Constipation-Symptoms, IRLSSG-RS - International Restless Legs Syndrome Study Group Rating Scale, BMI - Body mass index, ROC - Receiver Operating Characteristic Curve, RBD – Rapid Eye Movement Sleep Behavior Disorders, RBDQ-HK – Rapid Eye Movement Sleep Behavior Disorder Questionnaire – Hong Kong version, RBDSQ – Rapid Eye Movement Sleep Behavior Disorder Screening Questionnaire
