# Supplementary material for: BDNF and GDNF in Parkinson’s Disease: Associations with Clinical Features, Disease Course, and Progression—A Systematic Review
Source: Mol Neurobiol. 2026 Feb 16;63(1):440. doi: 10.1007/s12035-025-05649-z (PMC12909441; doi:10.1007/s12035-025-05649-z)
Supplement: Supplementary file 8 — (15.9 KB DOCX) [file 12035_2025_5649_MOESM8_ESM.docx]

Online Resource (Suppl. Table 8) Overview of studies assessing the associations between BDNF and GDNF levels and metabolic effects in Parkinson’s disease.

| **Number** | **Reference, year** | **Neurotrophin** | **Study groups: n** | **Assessed parameter** | **Summary of results** |
| --- | --- | --- | --- | --- | --- |
| 1 | Korkmaz et al. 2024 [42] | BDNF | PD:15 | LPS, Na+, K+, Ca2+, CRP, creatinine, LDH | BDNF was not significantly correlated with any of the assessed laboratory parameters. |
| 2 | Alomari et al. 2022 [43] | BDNF | PD: 24 | TC, HDL, LDL, TriG, fasting blood glucose, TC/HDL ratio, LDL/HDL ratio, TriG/HDL ratio, TC/LDL ratio | There were significant differences between the low-BDNF and high-BDNF groups in PD patients consecutively in:  - TC (low vs. high: 5.1±0.5 vs. 4.4±0.8, p=0.007),  - HDL (low vs. high: 1.1±0.2 vs. 0.9±0.2, p=0.06),  - LDL (low vs. high: 3.6±0.4 vs. 2.7±0.7, p=0.001),  - TC/LDL (low vs. high: 1.4±0.6 vs. 1.6±0.2, p =0.01),  - TriG/HDL (low vs high: 15 ±0.7 vs. 3.5±2.3, p=0.04).  There was a significant correlation between BDNF and:  - TC (r=−0.40, p=0.05),  - HDL (r =−0.60, p=0.003),  - LDL (r=0.50, p=0.006),  - TriG (r=0.40, p=0.04),  - TC/HDL (r=0.40, p=0.06),  - TC/LDL (r=0.5, p=0.01),  - TriG/HDL (r =0.60, p=0.001).  There was no association between BDNF and blood glucose level. |
| 3 | Alomari et al. 2018 [44] | BDNF | PD: 28 | Blood flow, vascular resistance, venous capacitance, venous outflow | BDNF levels were reduced in PD patients. In PD, BDNF predicted 18.5% of the variance in post-occlusion blood flow (p<0.05), 22.0% in vascular resistance (p<0.05), and 24.1% in venous capacitance (p<0.05).  Stepwise regression showed BDNF explained 26% of post-occlusion vascular resistance (p=0.008) and 42% of venous capacitance (p=0.002). |
| 4 | Ekmekyapar et al. 2021 [26] | BDNF | PD-MCI: 36  PD with mild dementia: 19  PD with moderate dementia: 8 | Serum 25(OH) vitamin D levels | There was no significant relationship between vitamin D and BDNF levels. |

**Abbreviations:** PD - Parkinson’s disease, BDNF - Brain-derived neurotrophic factor, GDNF - Glial-derived neurotrophic factor, PD-MCI - Parkinson's disease with mild cognitive impairment, LPS - Lipopolysaccharide, CRP - C-Reactive Protein, LDH - Lactate Dehydrogenase, TC - Total Cholesterol, HDL - High-Density Lipoprotein Cholesterol, LDL - Low-Density Lipoprotein Cholesterol, TriG - Triglycerides
