# Supplementary material for: BDNF and GDNF in Parkinson’s Disease: Associations with Clinical Features, Disease Course, and Progression—A Systematic Review
Source: Mol Neurobiol. 2026 Feb 16;63(1):440. doi: 10.1007/s12035-025-05649-z (PMC12909441; doi:10.1007/s12035-025-05649-z)
Supplement: Supplementary file 9 — (15.9 KB DOCX) [file 12035_2025_5649_MOESM9_ESM.docx]

Online Resource 9 (Suppl. Table 9) Overview of studies assessing the associations between BDNF and GDNF levels and confounding factors in Parkinson’s disease.

| **Number** | **Reference, year** | **Neurotrophin** | **Study groups: n** | **Summary of results** |
| --- | --- | --- | --- | --- |
| 1 | Huang et al. 2021 [24] | BDNF | PD with RLS: 53  PD without RLS: 196 | There was no significant correlation between BDNF and LEDD in either of the PD groups. |
| 2 | Roy et al. 2021 [7] | BDNF | PD: 27 | BDNF correlated negatively with inflammatory markers:  -IL-6 (r= -0.844, p=0.001)  -TNF-alpha (r= -0.846, p=0.001)  BDNF increased with the increase of IL-10 (r=0.966,p 0.001). |
| 3 | Huang et al. 2019 [10] | BDNF | PD: 28 | BDNF level in peripheral blood lymphocytes positively correlated with L-DOPA medication doses (r=0.548, p=0.003).  Patients with L-DOPA medication presented higher BDNF compared to those without L-DOPA treatment. |
| 4 | Huang et al. 2018 [23] | BDNF | PD: 60 | BDNF level increased with the higher doses of L-DOPA treatment (r=0.864, p<0.001). |
| 5 | Ventriglia et al. 2013 [28] | BDNF | PD: 30 | BDNF serum level was higher in patients treated with stabilizers/antiepileptics (p=0.009) and L-DOPA (p<0.001).  It was reduced in patients treated with benzodiazepines (p=0.020). BDNF levels were increased compared to healthy controls (p=0.045); however, due to all PD patients receiving L-DOPA, it was impossible to compare between PD with and without treatment. |
| 6 | Ziebell et al. 2012 [45] | BDNF | Patients with positive DAT-scan results: 21 | There was a significant correlation between serum BDNF levels and striatal neurodegeneration expressed as striatal [123I]PE2I DAT binding (linear regression: p<0.01; R2 =0.36). |
| 7 | Scalzo et al. 2010 [6] | BDNF | PD: 47 | There was no difference in BDNF serum levels between PD patients treated or not treated with L-DOPA (p=0.461). |

**Abbreviations:** PD – Parkinson’s disease, BDNF – Brain-derived neurotrophic factor, GDNF – Glial-derived neurotrophic factor, RLS – Restless legs syndrome, LEDD – Levodopa equivalent daily dose, IL – Interleukin, TNF-alpha – Tumor necrosis factor-alpha, DAT – Dopamine transporter, [123I]PE2I – radioligand used in DAT imaging.
